# Modeling, Evaluation, and In Vivo Estimation of Muscle Cell Diameter With the Random Permeable Barrier Model: Correlation With Subject Characteristics and Isometric Torque

**DOI:** 10.1002/nbm.70233

**Published:** 2026-01-20

**Authors:** Martijn Froeling, Roosmarijn Brenninkmeijer, Danny R. van der Woude, Bart Bartels, Linda Heskamp

**Affiliations:** ^1^ Center for Image Sciences, Department of Imaging and Oncology University Medical Center Utrecht Utrecht Netherlands; ^2^ Child Development and Exercise Center, Wilhelmina Children's Hospital University Medical Center Utrecht Utrecht Netherlands

**Keywords:** cell diameter, cross‐sectional evaluation, diffusion tensor imaging, healthy volunteers, muscle force, random permeable barrier model, simulations, skeletal muscle

## Abstract

The random permeable barrier model (RPBM) links the time‐dependent behavior of water diffusion in muscle tissue to its microstructure enabling estimation of average muscle cell diameter and membrane permeability. While RPBM has gained traction, few studies have examined the stability and limitations of the fitting process. Moreover, the added value of RPBM‐derived parameters compared with conventional diffusion tensor imaging metrics, and their relationship to subject characteristics and muscle function in healthy populations, remains underexplored. In this study, we comprehensively evaluated the RPBM by analyzing its forward behavior through simulations and its inverse behavior through model fitting. We further quantified muscle cell diameters across six muscle groups in 100 healthy adults and investigated how the derived parameters relate to DTI metrics, subject characteristics, and isometric muscle torque. The simulations showed that similar RPBM signals can arise from multiple parameter combinations and that the most stable estimates of cell diameter and membrane permeability were achieved by constraining τ and carefully selecting of $D_{0}$, with the best performance obtained when $D_{0}$ was fixed at 0.9 the axial diffusivity at long diffusion times. In vivo, muscle cell diameter differed across all muscle groups, and sex emerged as a strong determinant, with men exhibiting consistently larger cell diameters than women, independent of height and weight. However, no significant correlation was observed between peak torque and either cell diameter or membrane permeability. In conclusion, this study provides a comprehensive assessment of RPBM in healthy muscle and highlights its potential as a complementary tool to traditional diffusion metrics, particularly for studies of muscle health, development, and pathology, provided that its modeling limitations are carefully addressed.

## Graphical abstract

We evaluated the random permeable barrier model (RPBM) through simulations, model fitting, and in vivo analysis of 100 healthy adults. Simulations showed that stable estimates of muscle cell diameter and membrane permeability require constraining *τ* and fixing *D*_0_ at ~0.9 of axial diffusivity at long diffusion times. In vivo, muscle cell diameter varied across muscle groups and was strongly influenced by sex, while no correlation with muscle torque was found, highlighting RPBM's potential as a complementary diffusion metric.

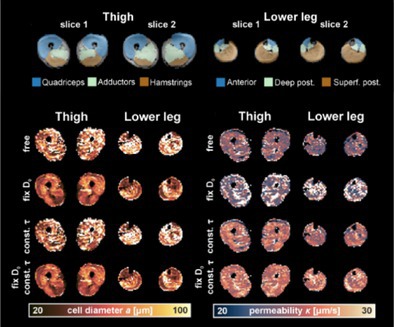

**Abbreviations**
AD: axial diffusivity
BMI: body mass index
CSA: cross‐sectional area
DTI: diffusion tensor imaging
FA: fractional anisotropy
IVIM: intravoxel incoherent motion
iWLLS: iterative weighted linear least squares
NLLS: nonlinear least squares
RD: radial diffusivity
REKINDLE: robust extraction of kurtosis indices with linear estimation
RPBM: random permeable barrier model
SE: spin echo
SNR: signal‐to‐noise ratio
STE: stimulated echo

## 1 Introduction

The random permeable barrier model (RPBM) links the time‐dependent behavior of water diffusion in muscle tissue to its underlying microstructure [1]. By modeling how semipermeable membranes restrict diffusion over time, RPBM enables the quantification of average muscle cell diameter and membrane permeability [2, 3, 4, 5]. Specifically, the model operates on the transverse component of the diffusion tensor, commonly referred to as radial diffusivity (RD), denoted $D_{⊥}\left(t\right)$, which is typically derived from diffusion tensor imaging (DTI). The RPBM provides a physiologically grounded model for how RD evolves over diffusion time due to cellular structures.

Previous studies have shown that RD is sensitive to muscle microstructure, particularly fiber diameter and organization. For example, changes in joint angle or voluntary contraction have been associated with measurable alterations in RD, consistent with shifts in fiber geometry [6, 7, 8]. Theoretical simulations further support this relationship, confirming RD's sensitivity to physiologically relevant changes in cell size [9, 10, 11]. Due to the dependency on muscle contraction, it is recommended to perform measurements in a fixed joint position to ensure consistency.

Accurate estimation of RD is challenging, regardless of the acquisition method used. RD is highly sensitive to noise, which causes eigenvalue repulsion and misestimation of the second and third eigenvalues (*λ*₂, *λ*₃), particularly underestimation of *λ*₃, leading to a downward bias in RD [12, 13]. Moreover, in muscle, *λ*₂ and *λ*₃ are considered unequal, likely due to myofiber ellipticity, further complicating interpretation [14]. Partial volume effects from fat lower all eigenvalues [15], and increased perfusion due to intravoxel incoherent motion (IVIM) effects can elevate RD [16, 17]. Spontaneous or intentional muscle contractions cause signal voids, which, if uncorrected, lead to overestimated RD [18].

Given these challenges, RPBM modeling depends on reliable RD measurements across a range of diffusion times, including those well beyond the muscle's $T_{2}$ relaxation time. To enable these extended diffusion times, diffusion‐weighted data for RPBM are typically acquired using stimulated echo (STE) sequences rather than conventional spin echo (SE). STE enables longer diffusion times because it stores magnetization along the longitudinal axis, making it subject to $T_{1}$ rather than $T_{2}$ decay.

However, STE suffers from reduced signal‐to‐noise ratio (SNR) and even more sensitivity to spontaneous muscle contractions [19, 20]. Despite these disadvantages, STE‐based diffusion MRI is widely used in skeletal muscle imaging and has demonstrated robust performance in both the thigh and lower leg [21]. In particular, STE‐based diffusion imaging has been shown to improve fiber tractography by increasing fractional anisotropy (FA) at longer diffusion times [22]. Moreover, when paired with optimized acquisition parameters, STE‐based diffusion imaging provides good reproducibility despite its lower SNR [23, 24].

Time‐dependent evaluation of RD is a valuable marker of microstructure, as demonstrated in mice investigating aging or Duchenne muscular dystrophy [25, 26], and in humans evaluating changes in Duchenne muscular dystrophy [27]. Time‐dependent change of RD can be interpreted more specifically using models such as the RPBM [3, 4]. Initial applications of RPBM modeling demonstrated its ability to estimate muscle fiber size and permeability in healthy subjects and muscle disease [2, 5]. In vivo studies have linked RPBM‐derived parameters to age‐ and disease‐related structural changes. For instance, RPBM has revealed decreasing surface‐to‐volume (SV) ratio, increasing cell diameter, and declining permeability in growing or aging mice and during Duchenne muscular dystrophy disease progression [28]. Similarly, studies in older adult humans have shown decreased cell diameter [29]. Furthermore, in humans, RPBM has been used to detect muscle atrophy [30], characterize Becker muscular dystrophy [31], and assess post‐marathon recovery [32].

Although RPBM has seen increasing application, few studies have critically examined the stability and limitations of the RPBM fitting process [2, 30]. The inverse model, that is, parameter fitting, is sensitive to noise and model constraints. Therefore, various stabilization strategies have been proposed, including fixing the free diffusion coefficient ($D_{0}$) [2] or applying dictionary‐based fitting approaches [30]. Moreover, although the derived parameters have been linked to disease, their added value compared with conventional DTI metrics and their relationships to subject characteristics and muscle function in healthy populations are still underexplored. These normal values are needed to be able to interpret observations in other cohorts, such as those with neuromuscular disease or disuse, and for understanding changes in longitudinal follow‐up and intervention studies.

In this study, we provide a comprehensive evaluation of the RPBM, including its forward behavior, fitting stability, and in vivo applicability. We first use simulations to characterize the model's forward and inverse behavior, which is necessary to understand its stability and interpretability before applying it to human data. We then estimate muscle cell diameters and permeability across six muscle groups in 100 healthy adults to understand normative values in a large cohort. Finally, we examine how the RPBM‐derived parameters relate to DTI metrics, subject characteristics, and muscle torque to determine whether RPBM provides additional insight beyond standard diffusion measures. These contextual analyses help position RPBM relative to commonly used demographic and functional markers.

## 2 Theory

Although the randomly permeable barrier model (RPBM) has been extensively described in previous studies [2, 3, 4, 5], we provide a brief overview of the necessary theoretical background to interpret the modeling and microstructural estimates presented in this work. The two primary parameters of physiological interest are the membrane permeability κ and the cell diameter a. The RPBM does not estimate these parameters directly, but it enables their calculation from intermediate fitted variables as described in this section.

### 2.1 RPBM Model

The RPBM describes the diffusion time‐dependent restricted diffusion transverse to the muscle fibers, specifically modeling the RD derived from DTI. The RPBM is characterized by three parameters: the free diffusion coefficient $D_{0}$; the membrane permeability κ, which is assumed to be the same for all membranes; and the membrane SV ratio S/V, where S is the total membrane surface area and V is the sample volume. The average distance between restrictions or barriers, interpreted as the “cell diameter” a, is related to the SV ratio by a=2d·V/S. In this context, d=2 because the diffusion is modeled in two transverse dimensions.

For a model of semipermeable barriers arranged in 2D, the radial diffusion coefficient D at diffusion time t is defined as $D\left(t;D_{0},\zeta ,\tau \right)=D_{0}\cdot F\left(t/\tau ;\zeta \right)$, where $D_{0}$ is the free diffusion coefficient. The parameter ζ is a dimensionless term proportional to the membrane density, and τ is the characteristic time scale associated with a single membrane.

Using substitution parameter s=t/τ>0, the ratio between the diffusion time t and τ becomes dimensionless. With α=1−ζ, the tortuosity factor, the function $F\left(t/\tau ;\zeta \right)=F\left(s;\alpha \right)$ is then defined as:

$$\begin{array}{c} F\left(s;\alpha \right)=\frac{1}{\mathrm{\pi s}}\int_{\;0}^{\infty }\frac{e^{-\mathrm{sy}}}{y^{2}}\left(f\left(y;\alpha \right)+Ay^{1/2}+Cy^{3/2}\right)\mathrm{dy}+\frac{1}{\alpha }+\frac{2A}{\sqrt{\mathrm{\pi s}}}+\frac{B}{s}-\frac{C}{\sqrt{\pi }s^{3/2}} \end{array}$$

Where,

fyα=Im1/α+2iy+y1+1−α/i+y2−1

$$A=2\left(\sqrt{\alpha }-1\right)/\alpha^{2}$$

$$B=2\left(\sqrt{\alpha }-1\right)\left(\sqrt{\alpha }-2\right)/\alpha^{2}$$

$$C=\left(8{\left(\sqrt{\alpha }-1\right)}^{2}-\left(1-\alpha \right)\sqrt{\alpha }\right)/\alpha^{4}$$

This equation enables the estimation of $D_{0}$, ζ, and τ from a series of measured radial diffusivities at various diffusion times t. Accordingly, here, we define all derived biophysical parameters in terms of the fitted parameters $D_{0}$, ζ, and τ. The characteristic parameters of the model, S/V; the surface to volume ratio; and κ, the membrane permeability, are thus defined as:

$$S/V=d\;\zeta /I=2\zeta /\sqrt{D_{0}\tau },\text{with}\;I=D_{0}/2\kappa =\sqrt{D_{0}\tau }$$

$$\kappa =\sqrt{D_{0}/\left(4\tau \right)}$$

where I represents the effective thickness, a characteristic length scale over which the diffusion gradient across the membrane is perturbed. As noted earlier, the average distance between restrictions a relates to the SV ratio and can be interpreted as the average “cell diameter,” which is defined as:

$$a=2d\cdot V/S=2\sqrt{{\mathrm{\tau D}}_{0}}/\zeta$$

Two other time scales were also introduced, $\tau_{d}$, the diffusion time to traverse a typical “cell,” and $\tau_{r}$, the residence time, which is the time to escape a given “cell” in the case when the escape process is permeability limited. These are defined as

$$\tau_{d}=a^{2}/2D_{0}=2\tau /\zeta^{2}$$

$$\tau_{r}=a/2\kappa =2\tau /\zeta$$

### 2.2 RPBM Fitting

Fitting the RPBM model is challenging due to its sensitivity to noise and the risk of convergence to local minima when using nonlinear least squares (NLLS) gradient–based optimization methods. To avoid local minima, fitting can be performed using a stochastic dictionary–based approach proposed by Lemberskiy et al. [30] (github.com/NYU‐DiffusionMRI/RPBM), which allows for robust, non‐gradient‐based parameter estimation. With this method, a global minimum is obtained by performing multiple random grid searches, each using a unique random noise‐perturbed dictionary. After fitting the data to each dictionary, the final parameter estimates are taken as the median across all runs.

The two primary parameters of physiological interest are the membrane permeability κ and the cell diameter a. The permeability κ depends only on $D_{0}$ and τ, whereas a is a function of $D_{0}$, ζ, and τ. Since $D_{0}$ acts only as a scaling factor, it can be fixed to a known value (e.g., the axial diffusivity, AD) to stabilize the fitting procedure, as proposed by Fieremans et al. [2]. This simplification leads to a more stable estimation of the remaining parameters, where $\propto 1/\sqrt{\tau }$, and $a\propto \sqrt{\tau }/\zeta$. If both τ and $D_{0}$ are fixed or tightly constrained, the permeability κ becomes fixed or constrained as well, reducing the number of free parameters in the model. In that case, the cell diameter a is effectively controlled by ζ alone, that is, a∝1/ζ.

## 3 Methods

All simulations, data processing, model fitting, and analysis were performed using the QMRITools package for Mathematica (www.qmritools.com) [33, 34]. The RPBM model fitting and evaluation functions were originally implemented in version 4.2.3, while all analyses presented in this manuscript were performed using version 4.3.6. Statistical analyses were conducted in RStudio [35].

### 3.1 Simulations

We first performed simulations to investigate how the derived parameters a (cell diameter) and κ (membrane permeability) depend on the model parameters $D_{0}$, ζ, and τ. Synthetic data were generated by uniform sampling of the parameter space: $D_{0}\in \left[1.0,2.0\right]$ μm^2^/ms, $\tau \in \left[100,900\right]$ ms, and $\zeta \in \left[0.5,2.0\right]$. These parameter ranges encompass known literature values and were selected to explore the model space without imposing tissue‐specific constraints. We performed two analyses: (1) evaluation of the forward model (simulation) to assess how the input parameters affect the derived quantities a and κ and (2) evaluation of the inverse model (fitting) to assess the robustness of parameter estimation under noise.

#### 3.1.1 Forward Modeling

For the forward model evaluation, we simulated radial diffusivities at diffusion times $t_{\text{full}}$ = 10, 20, 50, 100, 200, 500, 1000, 2000, and 5000 ms. Additionally, we examined a subset of clinically feasible times: $t_{\text{clin}}$ = 30, 110, 310, and 710 ms. We analyzed how RD evolved over time with varying model parameters, τ and ζ, and visualized the distributions of the derived quantities a and κ using histograms and contour plots.

#### 3.1.2 Inverse Modelling

To evaluate the inverse model, we simulated 5000 synthetic diffusion signals using both the full ($t_{\text{full}}$) and clinical ($t_{\text{clin}}$) diffusion time ranges. Additive Gaussian noise was applied such that the SNR of $D\left(t\right)$ was ~30. For fitting, we used the median parameter estimate across 100 dictionary fits. Each dictionary contained 1000 samples drawn uniformly from $\zeta \in \left[0.1,3.0\right]$ and $\tau \in \left[100,900\right]$ ms, and each used an independent noise realization.

Parameter fitting was performed using four approaches: (1) all parameters free, (2) fixed $D_{0}$, (3) constrained range of $\tau \in \left[350,750\right]$ ms, and (4) fixed $D_{0}$ with constrained τ. The SNR of each dictionary is set to 50 to match the SNR characteristics of the acquired in vivo data, ensuring a realistic fit. Additionally, we intentionally fixed $D_{0}$ to either a high or low value (± 0.3 μm^2^/ms) to evaluate how misestimating $D_{0}$ affects parameter recovery. Finally, we examined how estimated parameters κ and a relate to DTI metrics (FA, AD, and RD) that follow from the same simulations. In this context, $D_{0}$ is assumed to approximate AD and is thus treated as equivalent.

### 3.2 RPBM Evaluation in Healthy Controls

#### 3.2.1 Subjects

In this study, RPBM data and maximum voluntary torque measurements were acquired in 100 subjects. All subjects provided written informed consent, and the study was approved by the Medical Ethics Committee NedMec (ref. NL307.041.22). Inclusion criteria were an age between 16 and 60 years and a self‐reported body mass index (BMI) of 18–30 kg/m^2^. Exclusion criteria were contraindications to MRI and a history of neuromuscular disease.

#### 3.2.2 Body Composition and Torque

Body composition was characterized by measuring height, weight, and BMI. Maximal muscle strength of the right thigh was measured as peak isometric knee extension and flexion torque in Newton meter (Nm) using a Biodex dynamometer (System 4 Pro, Biodex Medical Systems, Shirley, NY, United States, Software version: Rev. 4.63 or advantage BX v5.3.06). Participants performed three cycles of seated maximal voluntary isometric flexion and extension at a 90° knee angle (5 s per contraction with 5‐s rest intervals), and the average peak torque across the three contractions was used for analysis [36]. If the coefficient of variation between the three repetitions was > 15%, the test was repeated once to ensure reliability. The strength measurements were conducted within 1 month before or after the completion of the MRI scan.

MRI acquisition was conducted on a 3‐T MR scanner (Ingenia, Philips Medical Systems) with a 12‐channel posterior coil and a 16‐channel anterior coil. The gradient system was operated at a maximum amplitude of 45 mT/m and a slew rate of 120 mT/m/ms. MRI data were acquired bilaterally in two slices in the thigh and two slices in the lower leg. The slices were placed halfway along the femur and at one‐third of the tibia length from the knee. STE EPI DTI (STE‐DTI) was performed using 15 diffusion weighted volumes: three volumes at *b* = 200 s/mm^2^ using three orthogonal directions and 12 volumes at *b* = 500 s/mm^2^ using 12 unique gradient directions isotropically distributed over a half‐sphere. Data were acquired across four diffusion times ($t_{\text{clin}}$ = 30, 110, 310, and 710 ms). Low *b*‐values were omitted from the acquisition to minimize IVIM effects in the RD estimation [17]. The full *b*‐matrix, accounting for all imaging gradients, was used to ensure accurate tensor estimation [5]. Acquisition parameters were as follows, slice thickness: 12 mm; in plane acquisition resolution 5 × 5 mm^2^, slice gap: 48 mm, TE/TR: 42/2000 ms, and SENSE factor: 2.4. Triple fat suppression was used, combining SPAIR, gradient reversal, and a custom pre‐pulse for olefinic fat suppression. The total acquisition time for the RPBM acquisition was 320 s (160 s per muscle region), corresponding to 40 s per diffusion time per muscle region with the same TR used for all diffusion times.

#### 3.2.3 Data Processing

Diffusion data were first denoised using a PCA‐based method [37, 38] and then corrected for eddy currents and subject motion [39, 40]. The DTI model was fitted per diffusion time using the iterative weighted linear least squares (iWLLS) method with REKINDLE outlier rejection [41, 42, 43, 44]. Outlier rejection is especially important since STE‐DTI is sensitive to signal dropout due to muscle fasciculations [45, 46]. The SNR of the data was calculated using the noise sigma estimated from the PCA‐based denoising step.

To assess the relationship between AD and $D_{0}$ for in vivo data, RPBM fitting was first applied to the volume‐averaged RD values of voxels within each region, that is, the thigh and lower leg, before fitting. Both nonlinear least squares (NLLS) and dictionary‐based fitting methods were used. The resulting $D_{0}$ estimates were compared with the mean AD measured at diffusion times $t_{\text{clin}}$ > 50 ms, based on the assumption that AD at long diffusion times approximates free diffusion in the absence of membrane restrictions [2].

Based on these fitting results, the ratio between $D_{0}$ and AD was used to fix $D_{0}$ in subsequent voxel‐wise fitting. In this step, RD was estimated separately for each voxel across all diffusion times. The RPBM model was then fitted per voxel using dictionary lookup.

#### 3.2.4 Data Analysis

We quantified the estimated diffusion parameters, cell diameter, and permeability for six muscle groups: the quadriceps, adductors, and hamstrings in the thigh, and the anterior, the deep posterior, and superficial posterior compartments of the lower leg. The muscle segmentations were performed on out‐of‐phase Dixon data using a U‐Net–based automated algorithm provided in QMRITools and were subsequently registered to the DTI data using B‐spline registration. For each muscle group, the local cross‐sectional area (CSA) was calculated as the mean of both legs and both slices. The total global CSA of the thigh and lower leg was computed as the sum of the three muscle groups at each region.

#### 3.2.5 Statistical Analysis

Four statistical analyses were performed on the derived diffusion and RPBM parameters and the measured average peak isometric extension and flexion torque.

First, derived values of cell diameter and membrane permeability were correlated with DTI‐derived parameters (FA, RD, and AD) using LOESS‐smoothed scatter plots to visualize nonlinear trends [47].

Second, subject‐level differences between men and women were assessed using *t*‐test [35] for each biometric and strength variable, that is, age, weight, height, BMI, total CSA of the thigh and lower leg, and average peak torque for isometric extension and flexion.

Third, to evaluate determinants of muscle‐group MRI parameters, linear mixed‐effects models [48] (level 1: muscle group, level 2: subject) were fitted for each outcome (a, κ, local CSA, FA, AD, RD) with age, weight, height, sex, BMI, and local CSA as fixed effects, and subject ID as a random intercept. The local CSA was omitted as a fixed effect when it was the outcome. All six muscle groups were included as a fixed factor, and their overall effect was tested using a Type‐III *F*‐test. When significant, Bonferroni‐corrected pairwise comparisons between muscle groups were performed using estimated marginal means.

Finally, for selected muscle groups, associations between average peak isometric extension and flexion torque and the MRI‐derived parameters were evaluated using muscle group specific multiple linear regression models [35]. These models included torque as the outcome and the MRI parameters as the primary predictor and were adjusted for sex and age, with the local CSA included as an additional covariate unless CSA was the outcome variable itself. Left–right differences of the MRI‐derived parameters were assessed using a mixed‐effects model with side as a fixed effect and subject as a random intercept. If no significant side effect was present, data from both legs were pooled for the torque analyses. The effect of adding sex, age, and CSA on model performance was evaluated by comparing models with an *F*‐test, with model improvement quantified via the change in Akaike information criterion (ΔAIC).

## 4 Results

### 4.1 Simulations

The simulations were performed to illustrate how each parameter uniquely shapes the time‐dependent behavior of RD and how well these effects can be recovered using model fitting. In these simulations, τ reflects membrane exchange time, ζ reflects membrane density, and $D_{0}$ reflects free diffusivity.

#### 4.1.1 Forward Modeling

**FIGURE 1:**
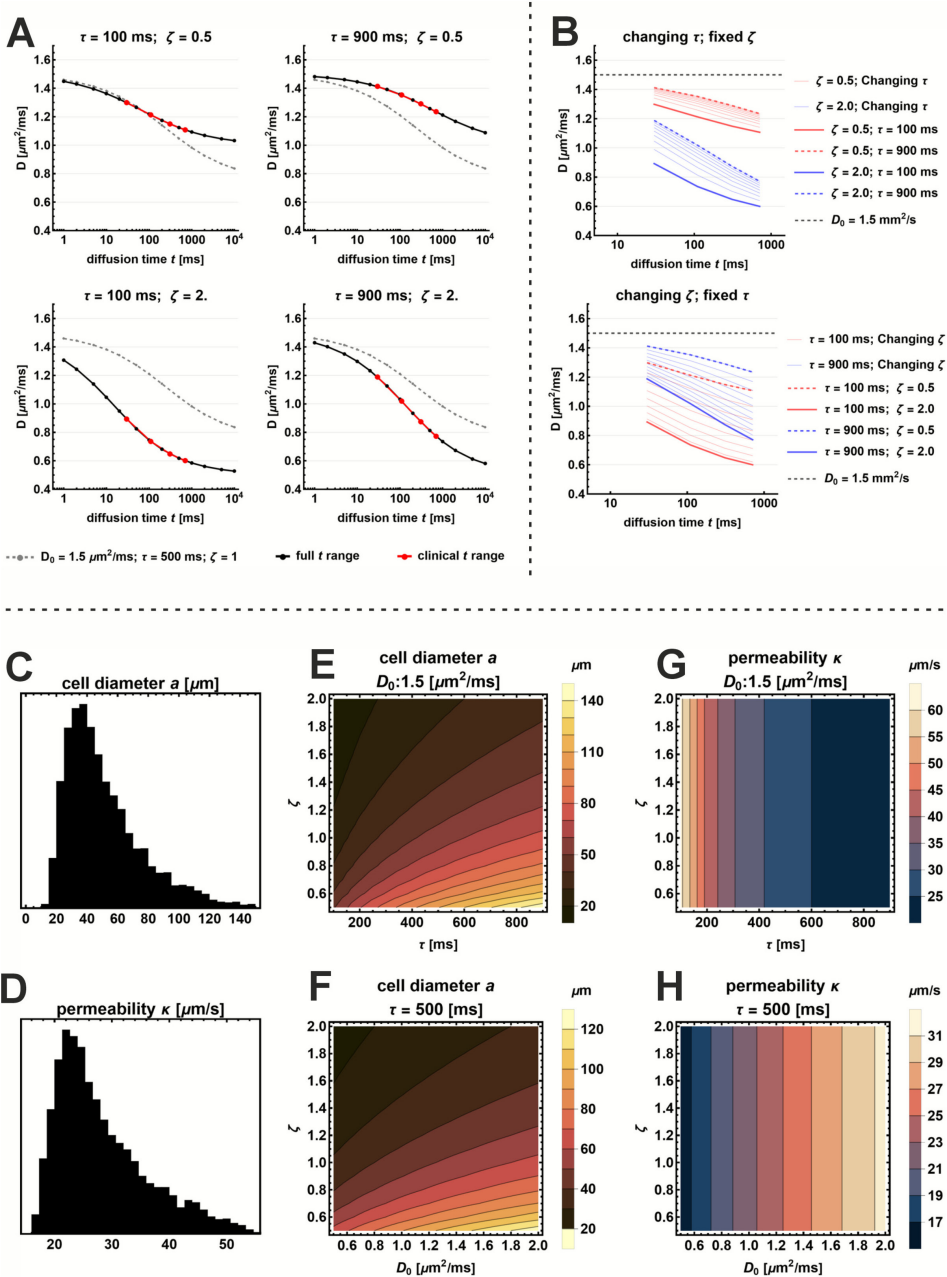
Forward model simulations illustrating how diffusion time dependence and model parameters affect derived microstructural metrics. (A) Simulated diffusion‐time‐dependent signal $D\left(t\right)$ for different τ and ζ values. Curves are shown for the full diffusion time range (black) and the clinically used range (red). (B) Impact on $D\left(t\right)$ of varying τ with a fixed ζ (top) and varying ζ with fixed τ (bottom). (C–D) Distributions of cell diameter a and permeability κ resulting from the noise free forward model when D₀, τ and ζ are all sampled from uniform distributions. (E–F) Simulated cell diameter as a function of τ and ζ, and as a function of ζ and D₀. (G–H) Simulated permeability as a function of τ and ζ, and of D₀ and ζ.

Figure 1 A shows the results of the forward model evaluation. For increasing values of ζ, the drop in RD from the free diffusivity $D_{0}$ to the long‐time limit $D_{\infty }$ becomes more pronounced, reflecting stronger restriction due to higher membrane density. Conversely, increasing τ shifts the diffusion time at which RD begins to drop. For very long diffusion times (t > 5000 ms), RD approaches a nonzero steady state $D_{\infty }$, determined by the combined effect of ζ and τ.

Within the clinically feasible diffusion time range (Figure 1B, top right), increasing ζ primarily modulates the slope and extent of the RD drop. A higher value of ζ leads to a faster and larger signal decay. In contrast, increasing τ reduces the drop in RD within the same time window, since less exchange occurs over the limited period. Figure 1B, bottom right, shows that when both τ and ζ are low (ζ < 1 and τ < 400) or both are high (ζ > 1 and τ > 500), the resulting RD curves overlap substantially. This shows that different parameter combinations can produce a very similar signal decay, particularly within the clinically relevant diffusion time range.

Figure 1C,D illustrate how the derived parameters a (cell diameter) and κ (membrane permeability) depend on the fitted model parameters τ, ζ, and $D_{0}$. Histograms of other parameters can be seen in Figure S1. When τ, ζ, and $D_{0}$ are sampled uniformly within physiologically plausible ranges, the resulting distributions of a and κ resemble skewed normal distributions. Notably, the range and variability of these derived parameters are consistent with those expected in vivo, despite being obtained through uniform random sampling across a broad range of τ, ζ, and $D_{0}$.

Figure 1E,F show that extreme values of a (very small or very large cell diameters) are only produced by narrow combinations of input parameters. In contrast, cell sizes in the physiologically realistic range of 40–80 μm can be obtained from a much broader range of parameter combinations. Figure 1G,H show the estimation of permeability κ that depends exclusively on accurate values of τ and $D_{0}$.

#### 4.1.2 Inverse Modeling

**FIGURE 2:**
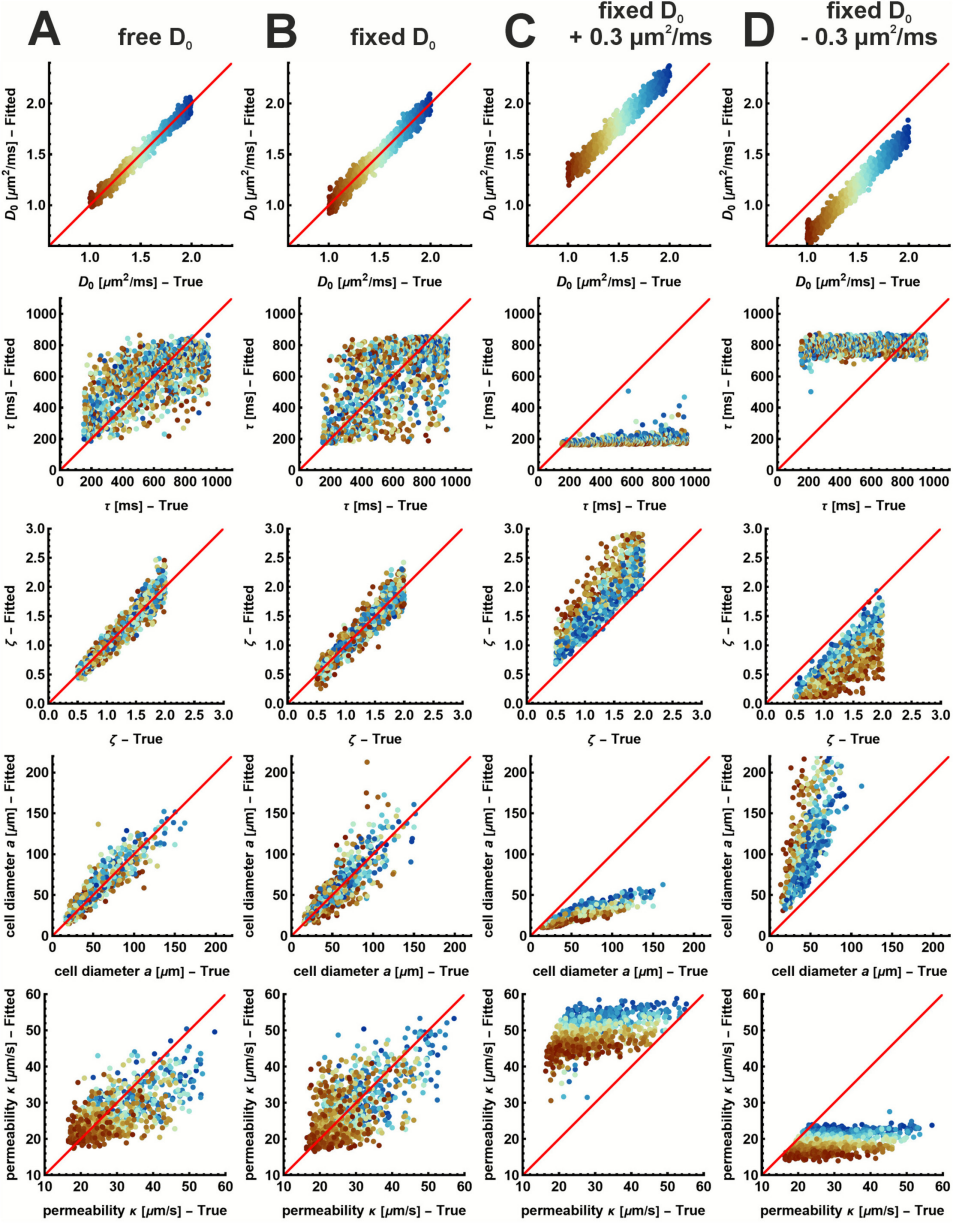
Parameter estimation accuracy for different fitting strategies using all diffusion times ($t_{\text{full}}$). Scatter plots compare fitted versus true values for all model parameters across 5000 simulated signals using four fitting strategies (columns): all free (A), fixed $D_{0}$ (B), and $D_{0}$ fixed incorrect (C–D). Rows show parameters $D_{0}$, τ, ζ, cell diameter a, and permeability κ. Red line indicates the identity line. The points are colored according to their value of $D_{0}$.

All results presented in Figure 2 are for diffusion times $t_{\text{full}}$ and without constraining τ. Corresponding results for fitting with constrained τ using $t_{\text{full}}$ and both fitting methods with the clinically feasible diffusion times $t_{\text{clin}}$ are given in Figures S2, S3, and S4. Overall, the findings were consistent across both diffusion time ranges, $t_{\text{full}}$, and $t_{\text{clin}}$.

Fitting the RPBM model with all parameters free ($D_{0}$, ζ, and τ) resulted in a slight overestimation of the cell diameter a and a considerable underestimation of permeability κ (Figure 2A). Fixing $D_{0}$ to the correct value reduced this bias and stabilized the estimation of a and κ, although the estimation of τ remained unstable (Figure 2B).

However, fixing $D_{0}$ too high resulted in a strong underestimation of a and an overestimation of κ, with τ shifting to the lower limit and ζ being overestimated (Figure 2C). Conversely, fixing $D_{0}$ to a value that was too low led to the opposite pattern (Figure 2D). Constraining τ to a narrow range slightly improved the accuracy of a regardless of whether $D_{0}$ was fixed or free (Figure S2A,B). Furthermore, constraining τ limited the misestimation of κ when $D_{0}$ is wrongly defined (Figure S2C,D).

Quantitative comparisons across fitting methods are summarized in Table 1. Fixing $D_{0}$ or constraining τ improved RMSE and *R* ^2^ for cell diameter a, with the lowest RMSE and *R* ^2^ observed when $D_{0}$ was fixed and τ was constrained. However, while constraining τ improved permeability κ estimates when $D_{0}$ was misestimated, it slightly worsened the accuracy when $D_{0}$ was correct.

**TABLE 1:** Fitting accuracy for estimated cell diameter a and membrane permeability κ under different model constraints for simulated data using $t_{\text{full}}$ and parameters sampled from $D_{0}\in \left[1.0,2.0\right]$ μm^2^/ms, $\tau \in \left[100,900\right]$ ms, and $\zeta \in \left[0.5,2.0\right]$.

Cell diameter a (μm)			
Fitting method	Mean ± STD (range)	RMSE	*R* ^2^
Free	2.04 ± 9.06 (−41.35 to 55.85)	9.29	0.86
Fix $D_{0}$	1.00 ± 14.21 (−67.00 to 112.82)	14.24	0.68
Fix $D_{0}$ too high	−27.53 ± 17.77 (−102.68 to −1.49)	32.77	−0.71
Fix $D_{0}$ too low	110.06 ± 106.67 (9.81 to 457.28)	153.26	−36.32
Free‐constr. τ	1.44 ± 9.47 (−37.90 to 64.44)	9.58	0.85
Fix $D_{0}$‐constr. τ	1.58 ± 12.42 (−52.36 to 157.43)	12.52	0.75
Fix $D_{0}$ too high‐constr. τ	−21.91 ± 15.94 (−94.66 to 2.20)	27.09	−0.17
Fix $D_{0}$ too low‐constr. τ	99.40 ± 97.56 (5.56 to 417.91)	139.27	−29.82
Permeability κ (μm/s)			
Fitting method	Mean ± STD (CI)	RMSE	*R* ^2^
Free	−1.75 ± 5.94 (−24.18 to 15.88)	6.20	0.41
Fix $D_{0}$	−0.10 ± 6.91 (−25.45 to 22.86)	6.91	0.27
Fix $D_{0}$ too high	20.57 ± 6.91 (−0.96 to 34.03)	21.70	−6.23
Fix $D_{0}$ too low	−9.11 ± 7.46 (−34.74 to 1.12)	11.78	−1.13
Free‐constr. τ	−2.31 ± 6.86 (−26.01 to 9.43)	7.24	0.20
Fix $D_{0}$‐constr. τ	−1.85 ± 6.62 (−26.37 to 10.76)	6.88	0.27
Fix $D_{0}$ too high‐constr. τ	5.19 ± 7.29 (−19.42 to 15.95)	8.95	−0.23
Fix $D_{0}$ too low‐constr. τ	−6.94 ± 7.51 (−31.98 to 3.53)	10.22	−0.60

**FIGURE 3:**
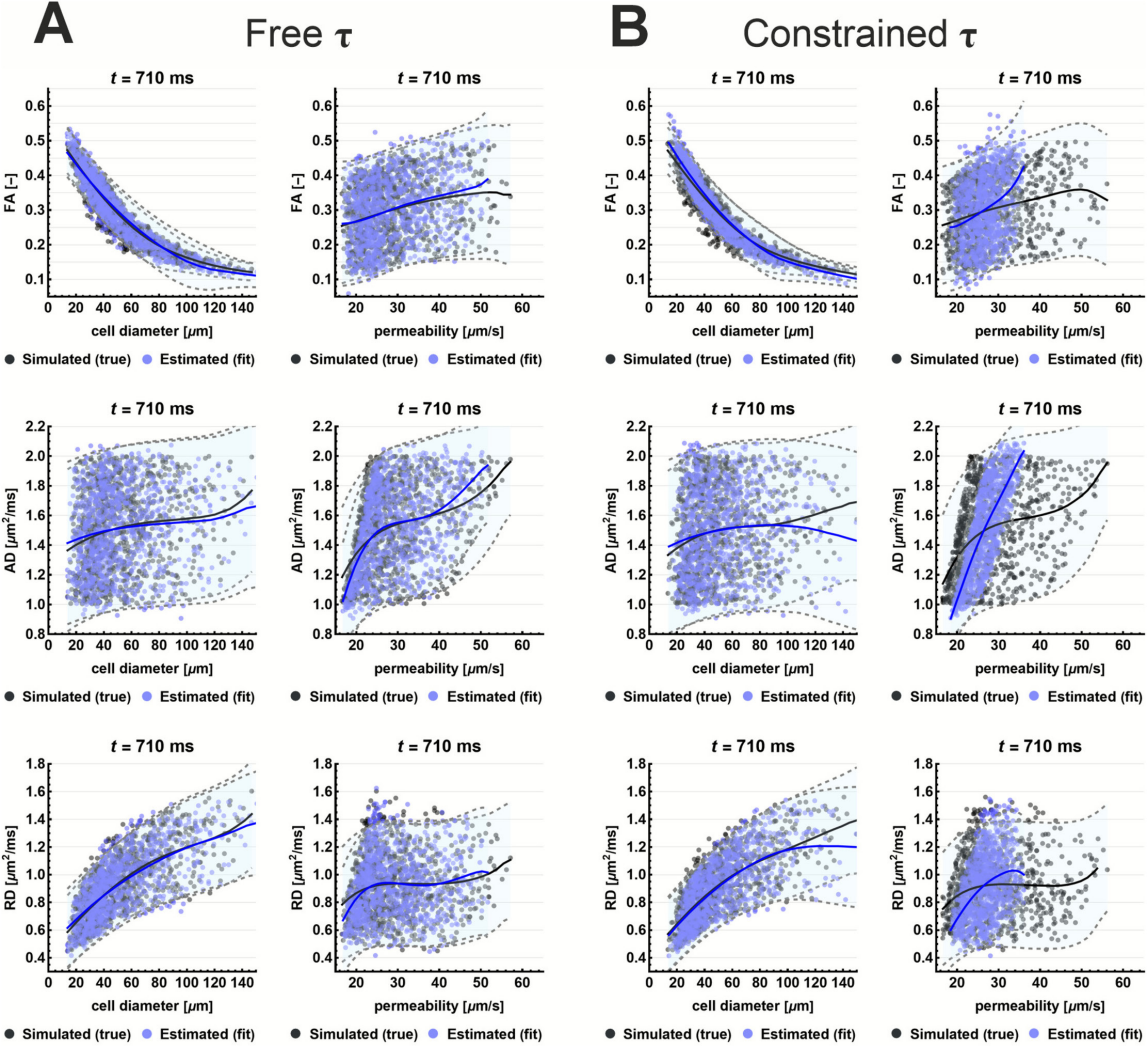
Simulated correlations between DTI metrics (FA, AD, RD) and microstructural parameters (cell diameter a, permeability κ) at *t* = 710 ms. Panels show both true (simulated) and fitted (estimated) values for fitting with τ free (A) and constrained (B). An inverse relationship is observed between FA and cell diameter, primarily driven by RD. Permeability correlates with AD, especially when *τ* is fixed, due to model constraints linking *κ* and *D*₀*.* Solid lines represent LOESS fits; shaded areas show 95% confidence intervals. Simulations are performed with $D_{0}\in \left[0.5,2.0\right]$ μm^2^/ms, $\tau \in \left[100,900\right]$ ms, and $\zeta \in \left[0.5,2.0\right]$.

Simulated correlations with DTI parameters (Figure 3) showed a strong inverse relationship between FA and cell diameter a. Additionally, when τ was constrained, a spurious positive correlation between κ and AD emerged, consistent with theoretical expectations, as restricting τ couples κ more directly to $D_{0}$.

### 4.2 RPBM Evaluation in Healthy Controls

#### 4.2.1 Subject Characteristics

Of the 100 participants who successfully underwent MRI scanning, five were excluded due to failed torque measurements, resulting in a final dataset of 95 subjects with complete imaging and strength data. The average age of the included subjects was 35.6 ± 12.7 years (range: 16.0–60.0 years), and the time between the MRI and torque measurements was 9 ± 7 (−14 to 26) days. Further subject characteristics and average peak torque values are reported in Table 2. Men were taller and heavier than women (*p* < 0.001), generated higher isometric extension and flexion peak torques (*p* < 0.001), and had larger total muscle CSA (*p* < 0.001). No significant sex differences were observed for age or BMI.

**TABLE 2:** Subject characteristics and strength measurements shown as mean ± standard deviation (range). The reported *p*‐values indicate differences between men and women. Bold‐italic values indicate statistical significance (*p* < 0.05).

	All (*n* = 95)	Men (*n* = 48)	Women (*n* = 47)	*p* men versus women
Age (year)	35.6 ± 12.7 (16.0 to 60.0)	35.7 ± 12.1 (16.0 to 60.0)	35.4 ± 13.4 (17.0 to 59.0)	0.902
Weight (kg)	74.7 ± 10.4 (54.8 to 103.4)	80.4 ± 9.4 (58.9 to 103.4)	68.8 ± 8.0 (54.8 to 88.3)	***< 0.001***
Height (cm)	176.4 ± 9.1 (152.8 to 200.9)	182.3 ± 7.2 (168.4 to 200.9)	170.4 ± 6.6 (152.8 to 185.2)	***< 0.001***
BMI (kg/m^2^)	24.0 ± 2.7 (17.9 to 31.1)	24.2 ± 2.5 (17.9 to 29.3)	23.7 ± 2.8 (18.6 to 31.1)	0.405
Total CSA thigh (cm^2^)	136.0 ± 25.3 (82.8 to 193.3)	154.2 ± 19.6 (112.6 to 193.3)	117.3 ± 14.3 (82.8 to 162.8)	***< 0.001***
Total CSA lower leg (cm^2^)	78.9 ± 12.2 (53.9 to 130.4)	84.8 ± 12.4 (61.8 to 130.4)	72.8 ± 8.5 (53.9 to 97.2)	***< 0.001***
Av. peak torque isom. ext. (Nm)	153.7 ± 40.9 (54.3 to 295.3)	181.7 ± 35.7 (54.3 to 295.3)	125.0 ± 21.6 (65.5 to 174.9)	***< 0.001***
Av. peak torque isom. flex. (Nm)	81.8 ± 25.1 (35.0 to 144.6)	97.8 ± 21.8 (41.4 to 144.6)	65.4 ± 16.3 (35.0 to 114.8)	***< 0.001***

#### 4.2.2 DTI and RPBM Parametric Maps

**FIGURE 4:**
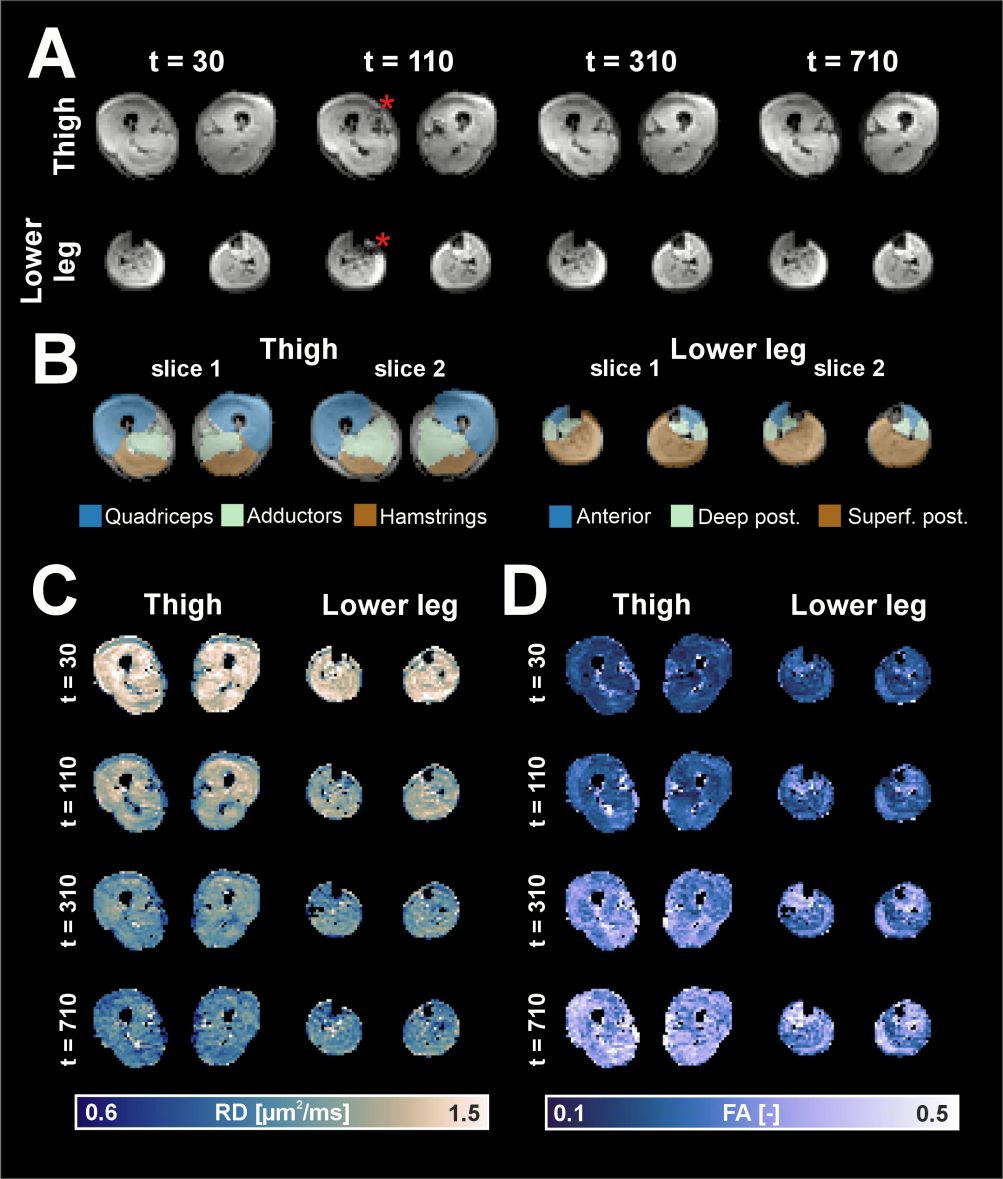
(A) STE‐DTI images across four diffusion times (t) show signal reduction from fat at longer diffusion times. Furthermore, at some time‐points, fasciculations can be observed (red asterisks). (B) Segmented muscle groups. (C‐D) RD and fractional anisotropy (FA) maps at corresponding diffusion times demonstrate diffusion time–dependent changes and regional variation across muscles.

Figure 4A shows representative data from the STE‐DTI acquisition across four diffusion times ($t_{\text{clin}}$ = 30, 110, 310, and 710 ms) in the thigh and lower leg. The average SNR of the data was 27.2 ± 3.3 (range: 19.1–34.2) for the thigh and 34.9 ± 4.5 (range: 23.5–44.3) for the lower leg. The STE sequence was sensitive to muscle fasciculations, visible as signal fluctuations in individual frames, as shown in t = 110 ms. Each dataset contained 120 slices. On average, 26 ± 24 (range: 2–154) fasciculations larger than four voxels were observed in the lower leg and 14 ± 13 (range: 0–85) in the thigh. The fat signal was reduced at longer mixing times due to rapid $T_{1}$ recovery during the mixing period. Muscle regions were segmented into six muscle groups for analysis, as shown in Figure 4B. As expected, RD decreased with an increasing diffusion time (Figure 4C). Corresponding changes were also observed in FA, which increased with diffusion time (Figure 4D). There was consistent, muscle‐specific spatial variation in AD, RD, and FA across diffusion times, with each muscle maintaining its characteristic pattern without irregular changes.

**FIGURE 5:**
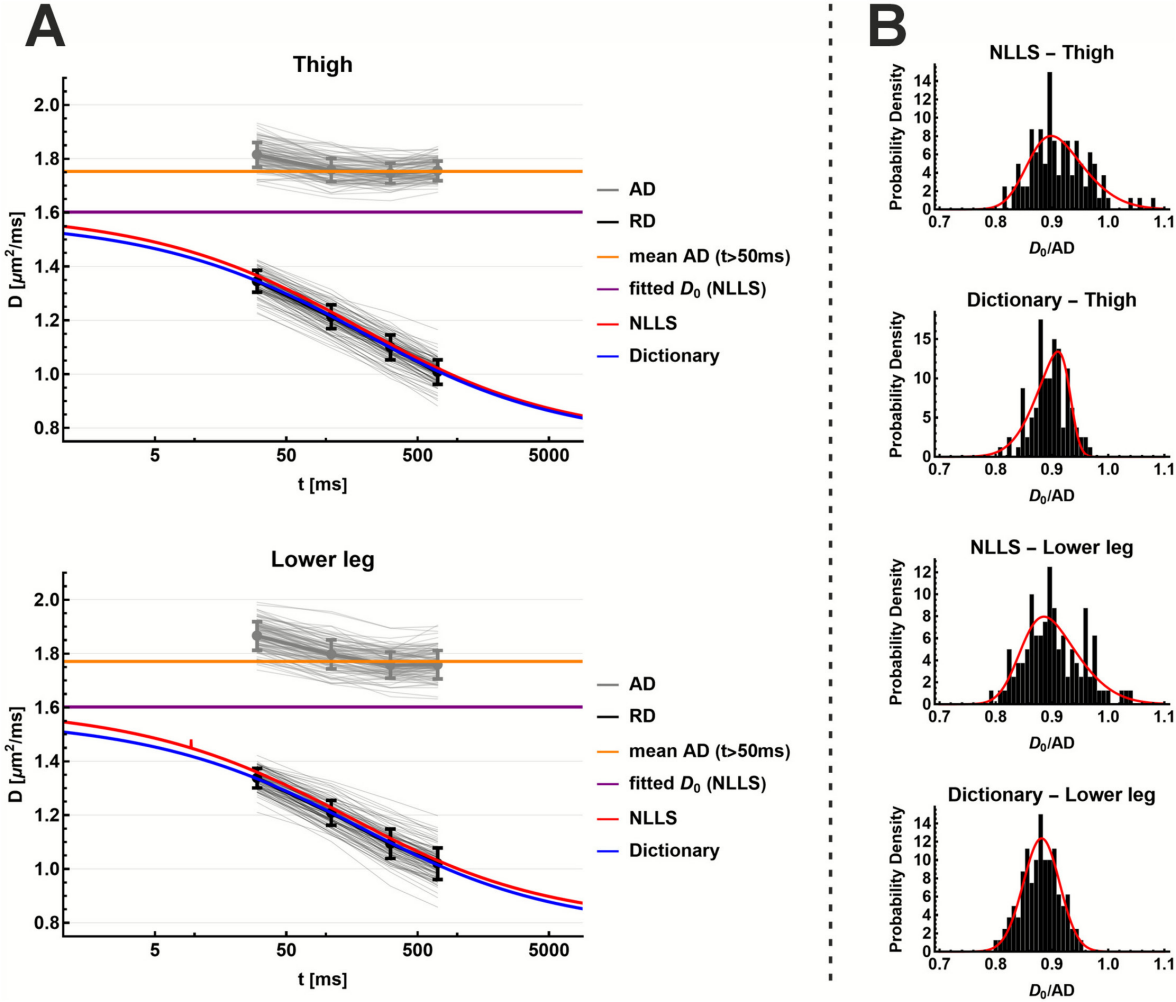
(A) RPBM fits to volume‐averaged radial diffusivity (RD) curves for the thigh (top) and lower leg (bottom). Thin gray and black lines show individual subject AD and RD values, respectively. The bold points with error bars indicate the group mean ± standard deviation. Group averaged model fits from NLLS (red) and dictionary‐based (blue) methods are overlaid. Mean axial diffusivity (AD, *t* > 50 ms) is shown in orange. The fitted D₀ from NLLS is shown in purple. (B) Histograms of the D₀/AD ratio across subjects per region for both fitting methods.

As shown in Figure 5A, both NLLS and dictionary‐based methods produced nearly identical fits to the volume‐averaged RD curves in the thigh and lower leg. All three fitted parameters ($D_{0}$, ζ, and τ) were comparable between methods (Table 3). The average fitting times per mean signal were 3292 ± 585 ms (range: 1742–5047 ms) for NLLS, 235 ± 28 ms (range: 212–451 ms) for the dictionary method, and 13 ± 2 ms (range: 11–26 ms) for the fixed $D_{0}$ dictionary method. Across subjects, fitted $D_{0}$ values were consistently around 90% of the mean AD measured at long diffusion times (t>50 ms), with similar distributions for both methods and regions (Figure 5B). Based on this consistency, $D_{0}=0.9\;\mathrm{AD}\left(t>50\right)$ was used as a fixed value in all subsequent voxel‐wise fits.

**TABLE 3:** Mean ± standard deviation (range) of RPBM fitting results ($D_{0}$, τ, and ζ) using average RD values per region across all subjects. Results are shown separately for thigh and lower leg using both NLLS and dictionary‐based fitting.

Muscle region	Fitting method	$D_{0}$ (μm^2^/ms)	τ (ms)	ζ
Lower Leg	NLLS	1.60 ± 0.08 (1.40 to 1.83)	434.11 ± 291.25 (111.00 to 999.86)	1.01 ± 0.20 (0.53 to 1.66)
	Dictionary	1.60 ± 0.08 (1.40 to 1.83)	430.37 ± 236.49 (169.79 to 871.41)	1.03 ± 0.20 (0.52 to 1.67)
Thigh	NLLS	1.60 ± 0.09 (1.42 to 1.89)	584.77 ± 328.35 (100.02 to 999.88)	1.12 ± 0.19 (0.52 to 1.77)
	Dictionary	1.60 ± 0.09 (1.42 to 1.89)	556.08 ± 262.13 (168.69 to 872.71)	1.11 ± 0.18 (0.54 to 1.76)

**FIGURE 6:**
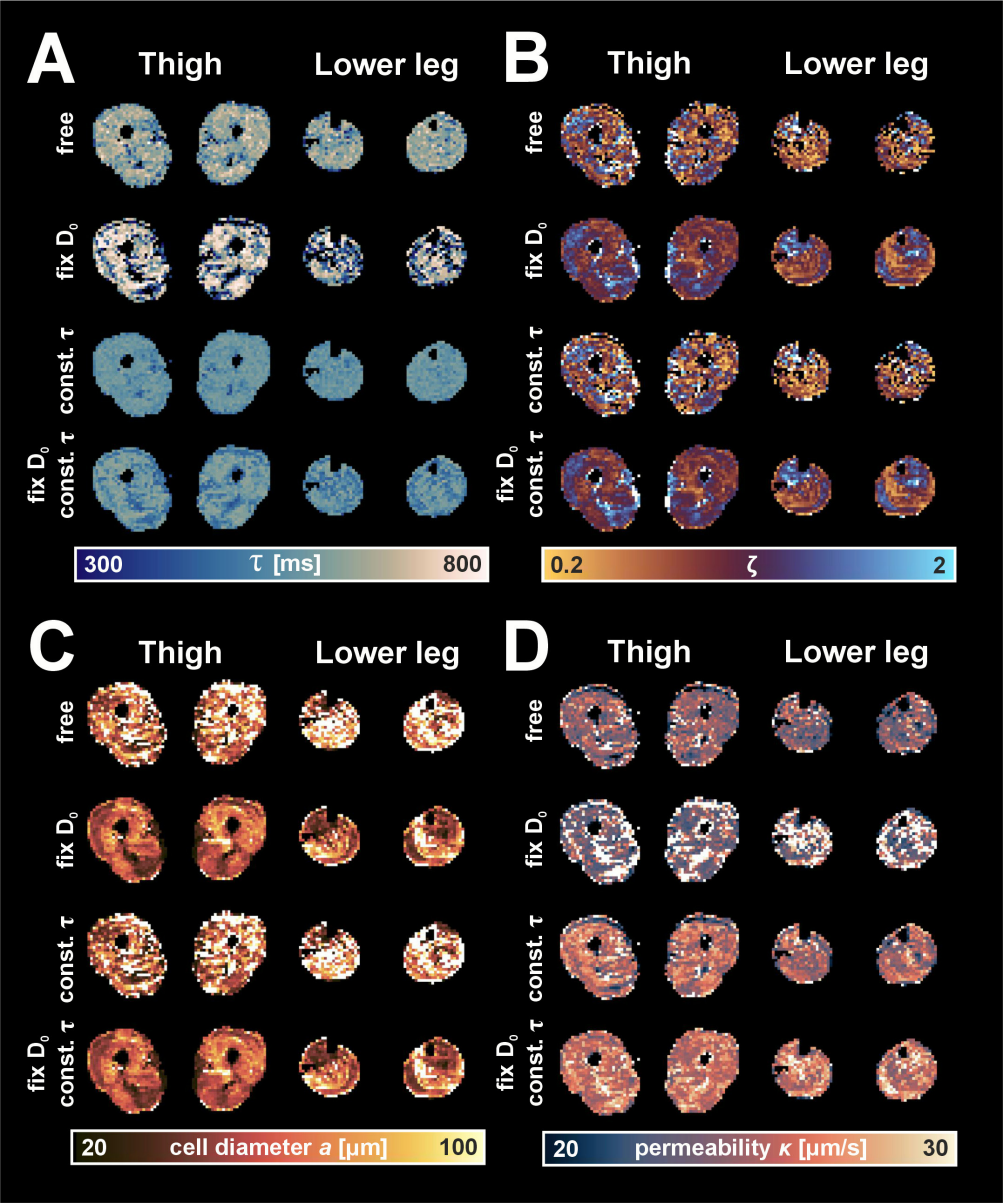
In vivo parameter maps using four fitting strategies: free fit, fixed D₀, constrained τ only and fixed D₀ with constrained τ. Maps for (A) τ, (B) ζ, (C) cell diameter a, and (D) permeability κ.

Figure 6 shows voxel‐wise maps of the RPBM parameters derived from in vivo fitting using different fitting constraints. The maps of the directly fitted parameters τ and ζ appear noisy and lacked anatomical structure, showing little correspondence with muscle boundaries (Figure 6A,B) when all parameters are free. Fixing $D_{0}$ partially stabilizes τ but drives it toward its upper limit, with ζ compensating accordingly. Constraining τ in addition to fixing $D_{0}$ markedly improves the homogeneity of both maps, reducing outliers and limiting parameter degeneracy. While fixing $D_{0}$ reduces noise in the diameter maps, κ remains spatially unstable (Figure 6C,D). Only when τ is also constrained do both a and κ appear more consistent with expected distributions. These in vivo results mirror the simulation findings, showing that errors in $D_{0}$ propagate through τ and ζ, ultimately biasing a and distorting κ.

#### 4.2.3 Correlation With DTI Metrics

**FIGURE 7:**
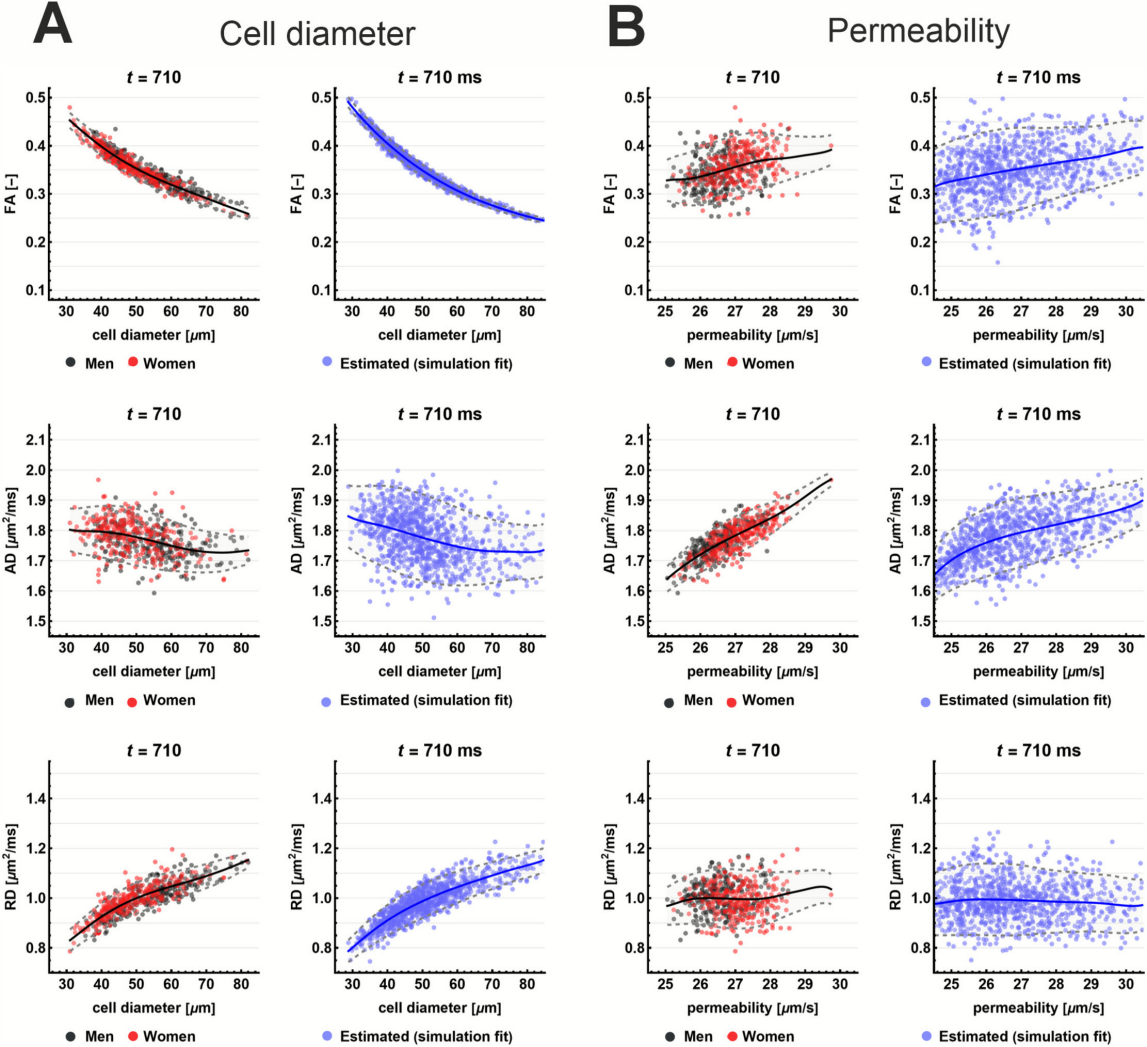
Relationship between DTI metrics (FA, AD, RD) and RPBM‐derived parameters (cell diameter, permeability) at *t* = 710 ms. The left columns show in vivo data for men (black; *n* = 288) and women (red; *n* = 282), where each dot represents a muscle group for a subject. The right columns show simulation derived DTI parameters for data sampled from $D_{0}\sim N\left(1.6,0.1\right)$ μm^2^/ms, $\tau \sim N\left(550,50\right)$ ms and $\zeta \sim N\left(1.2,0.2\right)$ in blue.

Figure 7 illustrates the in vivo relationships between RPBM‐derived microstructural parameters and DTI metrics at *t* = 710 ms. The same plots for all in vivo diffusion times are shown in Figures S5 and S6. Cell diameter a shows a strong inverse relationship with FA, which is most pronounced at long diffusion times and is driven by the corresponding decrease in RD (Figure 7A). Since D₀ was fixed and τ was constrained, permeability κ is primarily modulated by D₀, which approximates AD at long diffusion times. As a result, permeability and AD exhibit a strong linear relationship, matching simulations where the parameters are derived from data sampled from $D_{0}\sim N\left(1.6,0.1\right)$ μm^2^/ms, $\tau \sim N\left(550,50\right)$ ms, and $\zeta \sim N\left(1.2,0.2\right)$ (Figure 7B), which approximated the average fit results (Table 3).

#### 4.2.4 Effect of Sex and Muscle Region

**FIGURE 8:**
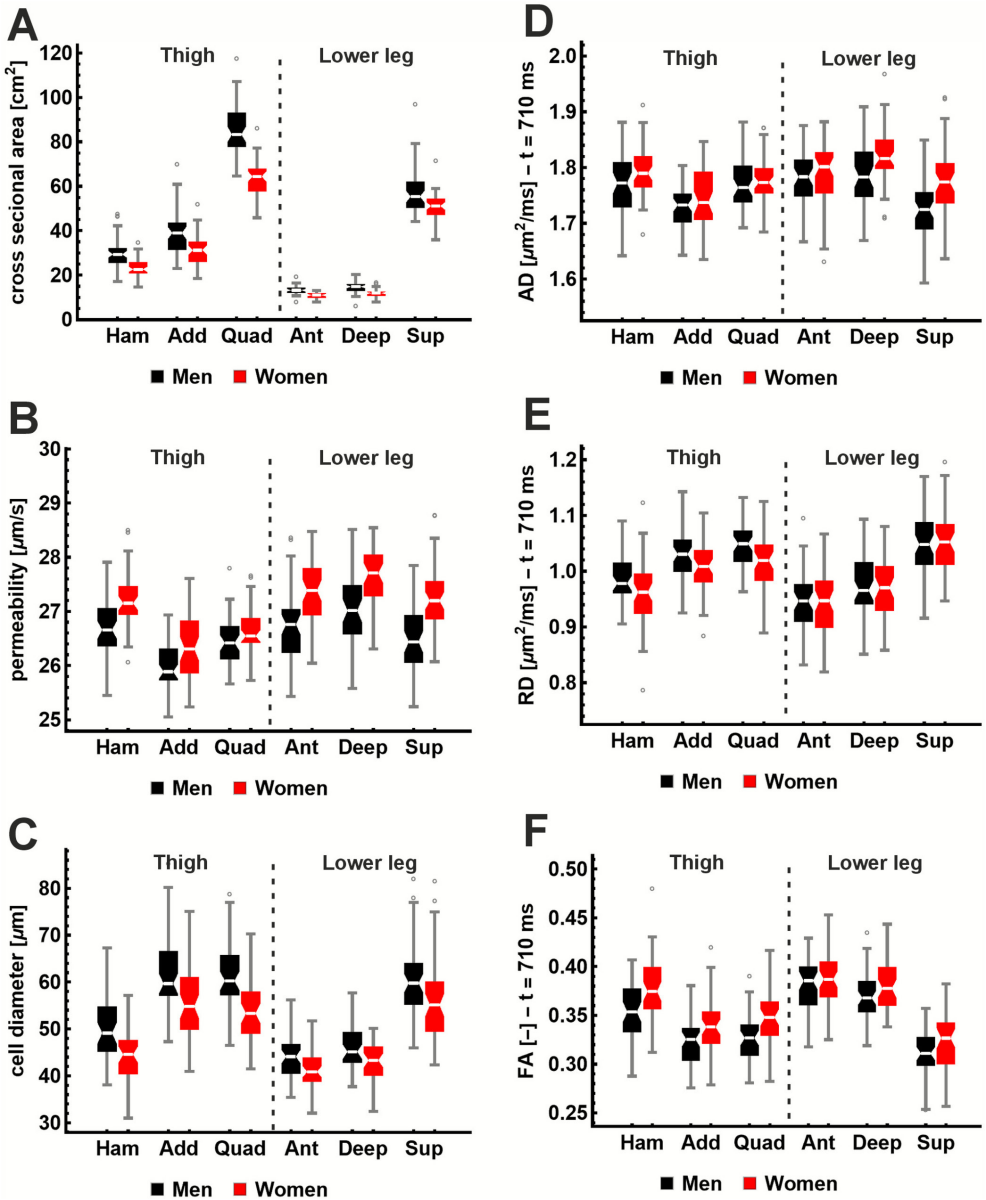
Box plots of cross sectional area (A), permeability (B), cell diameter(C), AD (D), RD (E), and FA (F) across six muscle groups in the thigh and lower leg, separated by sex (black = men, *n* = 48, red = women, *n* = 47).

Figure 8 shows the local CSA, cell diameter a and permeability κ, and DTI parameters AD, RD, and FA across muscle groups, separated by sex. The linear mixed‐effects model (Table 4A) showed that local CSA, cell diameter, permeability, and AD differed between sexes, with men exhibiting larger local CSA and cell diameters and lower permeability. All parameters also differed significantly between muscle groups. The exact differences are detailed in Table S1.

**TABLE 4:** (A) Linear mixed‐effects model results for associations between MRI‐derived parameters (CSA, 𝑎 𝜅, FA, AD, RD) and subject characteristics. Models included age, weight, height, sex, BMI, and CSA as fixed effects. (B) Associations between average peak isometric extension and flexion torque and the MRI‐derived parameters, adjusted for sex, age, and CSA. (C) The same torque associations as (B) without adjustment for sex, age, or CSA. Reported values represent the parameter estimate for the primary predictor with its corresponding *p*‐value in brackets. Bold‐italic values indicate statistical significance (*p* < 0.05).

	Local CSA (cm^2^)	*α* (μm)	*κ* (μm/s)	FA (‐)	AD (μm^2^/ms)	RD (m^2^/ms)
A Correlation with biometrics						
Sex (women ‐ Men]	***−6.377 (< 0.001)***	***−3.483 (0.007)***	***0.626 (< 0.001)***	0.010 (0.065)	***0.036 (< 0.001)***	0.002 (0.841)
Muscle group	***‐ (< 0.001)***	***‐ (< 0.001)***	***‐ (< 0.001)***	***‐ (< 0.001)***	***‐ (< 0.001)***	***‐ (< 0.001)***
Local CSA(cm^2^)	—	***0.090 (0.002)***	***0.006 (0.029)***	0.000 (0.126)	***0.001 (0.005)***	***0.001 (0.001)***
Age (years)	0.027 (0.316)	***0.147 (< 0.001)***	0.004 (0.192)	***−0.001 (< 0.001)***	0.000 (0.954)	***0.001 (< 0.001)***
Weight (kg)	***0.684 (0.048)***	0.036 (0.939)	−0.020 (0.620)	0.001 (0.797)	0.001 (0.827)	0.000 (0.956)
Height (cm)	−0.471 (0.110)	0.026 (0.948)	0.023 (0.513)	−0.001 (0.694)	0.000 (0.943)	0.001 (0.734)
BMI (kg/m^2^)	−1.131 (0.291)	0.223 (0.878)	0.021 (0.869)	−0.003 (0.635)	−0.007 (0.540)	0.001 (0.961)
B Correlation with isometric torque (corrected for local CSA + sex + age)						
Quadriceps ‐	***0.165***	−0.044	0.003	0.000	0.000	0.000
Extension	***(< 0.001)***	(0.084)	(0.140)	(0.262)	(0.123)	(0.876)
Adductors ‐	0.009	0.007	−0.001	0.000	0.000	0.000
Extension	(0.789)	(0.779)	(0.749)	(0.749)	(0.764)	(0.764)
Hamstrings ‐	***0.139***	0.038	−0.004	0.000	0.000	0.000
Flexion	***(< 0.001)***	(0.254)	(0.269)	(0.332)	(0.986)	(0.388)
Adductors ‐	0.081	0.049	−0.001	0.000	0.000	0.000
Flexion	(0.102)	(0.207)	(0.609)	(0.438)	(0.638)	(0.352)
C Correlation with isometric torque (no corrections)						
Quadriceps ‐ Extension	***0.264 (< 0.001)***	***0.057 (0.002)***	−0.002 (0.083)	***0.000 (0.028)***	0.000 (0.610)	***0.000 (0.011)***
Adductors ‐	***0.076***	***0.052***	***−0.004***	***0.000***	0.000	0.000
Extension	***(0.002)***	***(0.010)***	***(0.008)***	***(0.046)***	(0.571)	(0.201)
Hamstrings ‐	***0.165***	***0.127***	***−0.010***	***0.000***	0.000	***0.001***
Flexion	***(< 0.001)***	***(< 0.001)***	***(< 0.001)***	***(< 0.001)***	(0.113)	***(0.002)***
Adductors ‐	***0.158***	***0.111***	***−0.006***	***0.000***	0.000	0.000
Flexion	***(< 0.001)***	***(< 0.001)***	***(0.006)***	***(0.014)***	(0.588)	(0.064)

#### 4.2.5 Associations With Subject Characteristics

The relationships between the derived microstructural parameters and subject characteristics, evaluated using the linear mixed‐effects model, are summarized in Table 4A. The local CSA was associated with a, κ, AD, and RD, although the effect sizes were very small. Muscle CSA increased with increasing weight. Cell diameter increased with age (0.147 μm/year), and age also showed small effects on FA and RD. All other parameters were independent of CSA, age, weight, height, and BMI. Scatter plots of all relationships are shown in Figure S7.

#### 4.2.6 Relation to Muscle Torque

No left–right differences were observed for any parameters; therefore, the average of both sides was used in the statistical testing against the right‐leg muscle torque. No significant associations were found between cell diameter, permeability, or DTI parameters and isometric peak torque in any muscle group (Table 4B). The only significant correlations were quadriceps CSA with extension torque and hamstring CSA with flexion torque. When the linear mixed‐effects model was not corrected for sex, age, and CSA, many significant associations with force appeared (Table 4C). Although the covariates sex, age, or CSA did not all reach significance in every model, their inclusion consistently improved global model fit (*p* < 0.01; ΔAIC > 6) of the data, except when AD was the outcome. Scatter plots are provided in Figure S8.

## 5 Discussion

This study aimed to evaluate the behavior of the RPBM model and apply it to quantify muscle cell diameter and membrane permeability in healthy adults. We evaluated the model through simulations and demonstrated accurate estimation of cell diameter under constrained conditions. The in vivo data identified anatomical and sex‐related differences. Cell diameter and permeability differed between muscle groups, and men had larger cell diameters with lower muscle permeability compared with women.

The evaluation of the forward model showed that similar RPBM signals can arise from multiple parameter combinations, particularly when τ and ζ are both low or both high. Small variations in the signal due to noise or measurement error may lead to large differences in the fitted parameter values, highlighting the risk of degeneracy in the inverse problem. This degeneracy is a known limitation of the model and motivated the use of dictionary‐based approaches [30]. Additionally, when τ, ζ, and $D_{0}$ are randomly sampled from uniform distributions, the derived estimates for cell diameter and permeability remain within plausible physiological ranges due to inherent model constraints that bias toward average cell sizes and low permeability. This highlights a potential pitfall in interpreting seemingly reasonable results: A noisy or ill‐conditioned fit can still produce values of cell diameter a and permeability κ that appear realistic. Finally, while very short τ values cause a large increase in permeability, withing the physiological range of τ, modulation of permeability is primarily controlled by $D_{0}$. This underscores the importance of a good initial estimate or constraint on $D_{0}$ during fitting.

For the evaluation of the inverse model, the overall most accurate estimates of a and κ were obtained by constraining τ combined with a careful selection of $D_{0}$, although this balance is delicate. Fixing $D_{0}$ causes τ and ζ to compensate, with overestimation of $D_{0}$ leading to underestimation of a, and vice versa. Constraining τ stabilizes κ but restricts its dynamic range, with minimal effect on a estimation. Leaving τ unconstrained yields more accurate estimates of κ when $D_{0}$ is correctly specified [2], whereas constraining τ can partially mitigate errors in cell diameter estimation when $D_{0}$ is misestimated. Importantly, when $D_{0}$ is misestimated, the relative ranking of cell sizes across muscle regions is preserved. Both simulations with a wide range of diffusion times (1–5000 ms), and a clinically feasible range (30–710 ms), produced similar bias patterns. Overall, fitting remains challenging even in the absence of noise, highlighting the complexity of the inverse problem. Taken together with the proposed fitting methods, these results indicate a clear distinction: cell diameter can be robustly fitted by fixing $D_{0}$ and constraining τ, but permeability cannot.

Simulations with broad τ, ζ, and $D_{0}$ sampling revealed correlations between RD, FA, and cell diameter, consistent with theoretical expectations and prior DTI studies [6, 7, 9]. In vivo, the same relation was present but confined to a narrower range for τ, ζ, and $D_{0}$, as expected for healthy muscle. Both simulations and prior studies showed that FA increases with cell diameter, especially at longer diffusion times [1, 10, 11] enhancing contrast [27]. The RPBM improves the interpretability of DTI by translating the AD/RD ratio into explicit cell diameter estimates. However, fitting introduces complications: permeability, which should be independent of AD, became correlated due to fitting instability. When $D_{0}$ is misestimated, τ shifts toward dictionary boundaries, effectively fixing κ and making it linearly dependent on $D_{0}$ and thus AD.

Simulations showed that fixing $D_{0}$ to an appropriate value stabilized cell diameter estimates while limiting permeability variability. This effect was also observed in vivo. Voxel‐wise free fitting produced wide and unstable ranges for both cell diameter a and permeability κ, whereas fixing $D_{0}$ or constraining τ stabilized cell diameter estimates and reduced permeability variability. In vivo, RPBM fits to volume‐averaged radial diffusivities of the thigh and lower leg indicated that $D_{0}$ is best fixed at $0.9\;\mathrm{AD}\left(t>50\right)$. While the assumption that AD at long diffusion times approximates free diffusion may hold in theory [2], this limit is not reached within feasible diffusion times.

Regional analysis showed that muscle cell diameter a differed across all muscle groups. Although absolute estimates of a may be influenced by misestimation of $D_{0}$, the relative differences between muscles remain reliable. Overestimation of $D_{0}$ reduced a and attenuates contrast, whereas underestimation amplifies inter‐muscle differences (Figure 2C,D). Compared with FA, which also captures regional variation, cell diameter a provided clearer anatomical separation. Apparent differences in permeability across muscles were also observed but are likely driven by differences in ad rather than true physiological variation, given the instability of permeability fitting. The cell diameter values observed here were consistent with previous human studies [2, 5, 29, 30, 31, 32]. However, as shown here with simulations, the obtained cell diameters are naturally constrained between 25 and 100 μm by the RPBM. Reported values of permeability were much more variable, with most around 30 μm/s, but some skewed more to lower values below 20 μm/s [5, 29] and some as high as 100 μm/s [2, 31]. These variations and skewing to limits were also apparent in our simulations and in vivo data, depending on the fitting method and how parameters were fixed or constrained.

In vivo results showed that sex was a strong determinant of cell size, with men consistently exhibiting larger cell sizes than women even after adjusting for height and weight. Conversely, permeability was lower in men, though this finding should be interpreted with caution, as explained before. Across our cohort (16–60 years), we saw a very small positive effect of age on cell diameter of around 0.15 μm/year. This was mostly driven by an increase in diameter in the range 16–30 years, with values remaining stable from 30 to 60 years. While age was significantly associated with cell diameter but not local CSA, the small effect sizes for both parameters suggest that macrostructural and microstructural changes remained relatively stable across the age range of our cohort. The study by Malis et al. included a senior age group between 65 and 76 years and observed no significant differences compared with the young group regarding cell diameter [29]. Diffusion parameters differed by muscle group, age, and sex. The difference between muscle groups is well studied [49], and the age‐related trends align with previous observations by Cameron et al. [50]. Reports on sex dependence of diffusion parameters are conflicting [51, 52]. In our data, which was acquired at long diffusion times and corrected for CSA, only AD showed a significant correlation with sex. In general, men have larger cell diameters and larger CSA than women but can also generate higher peak torque for the same cell diameter, which can be explained by their larger CSA. All correlations between a, κ, FA, and RD and torque lost significance when correcting for CSA, age, and sex. While individual covariates were not significant for every outcome, their inclusion ensured a consistent adjustment framework across all analyses. This approach significantly improved the model fit (*p* < 0.01; ΔAIC > 6) for all outcomes except AD.

Previous studies reported correlations between RD and measures of muscle strength in smaller, more homogeneous cohorts. Klupp et al. observed positive correlations with paraspinal strength ratios [53], and Carpenter et al. associated RD with in vivo quadriceps twitch properties but not voluntary strength in a woman only cohort [54]. Scheel et al. showed a positive correlation between RD and maximal mechanical power of the Soleus in a male only cohort [55], and Mazzoli et al. found a small positive correlation of RD with muscle torque in the thigh [56]. Furthermore, Mazzoli et al. also found that combining CSA and RD improved muscle torque prediction compared with CSA alone [56]. While we also observed a univariate relation between RD and muscle torque, this disappeared after adjusting for sex, age, and CSA. Similar patterns were observed for cell diameter, permeability, and FA. Together with findings from Mazzoli et al., this suggests that in larger, mixed‐sex populations, the macroscopic CSA is the primary driving force for these relations, likely modulated by underlying microstructural parameters. Overall, these results emphasize the importance of accounting for confounding factors, such as sex, age, and CSA in interpreting structural and functional associations.

This study employed voxel‐wise RPBM fitting followed by ROI‐based averaging of the parameters to capture regional muscle microstructure. Alternatively, per‐muscle estimates can also be obtained by first averaging RD within the ROI and then deriving RPBM parameters. Hybrid strategies, such as ROI‐based averaging to stabilize fitting followed by localized refinement, are also feasible. While healthy muscle is relatively homogeneous, pathological conditions may introduce spatial heterogeneity that favors the more challenging voxel‐level analysis.

Reliable RPBM estimation critically depends on accurate RD measurements, and when $D_{0}$ is constrained, AD as well. Several factors can influence these diffusion metrics. RD is particularly sensitive to noise, perfusion, and artifacts [12, 13, 15, 16, 17, 46], and it correlates with transient muscle stretch [6, 7, 8]. In addition, RD arises from two distinct eigenvalues in muscle [12, 13, 14], while the RPBM assumes equal diffusivities in all directions. Prior work has shown that omitting this assumption and fitting $\lambda_{2}$ and $\lambda_{3}$ separately leads to substantial differences in the derived RPBM parameters [29]. Moreover, muscles differ in their intrinsic AD [49, 57, 58], so if the assumption that AD ≈ $D_{0}$ does not hold, derived parameters may be biased.

Measurement‐related factors also introduce variability. Tensor estimates based on a limited number of directions are susceptible to outliers [19, 24], and signal voids can artificially inflate RD, AD, and FA [18]. This pattern likely leads to an underestimation of a and an overestimation of κ. Even with fat suppression, residual fat contributes a small isotropic low‐diffusion water component that decreases RD and AD while increasing FA [15, 59], again biasing the RPBM parameters in the same direction. Muscle perfusion produces a different pattern of bias: It affects AD and RD unequally [16, 17], altering their ratio and FA, which tends to overestimate a and underestimate κ. Finally, physiological variation in muscle position, that is, passive shortening or elongation, change the apparent fiber diameter [9, 10, 11], introducing additional variability in a.

Each of these confounds is addressed here using high *b*‐values, triple fat suppression, robust outlier detection, and fixation of the foot to stabilize muscle position. Nevertheless, they cannot be fully eliminated, and the exact contribution of each factor requires further study. In general, these confounds add uncertainty, variability, or even systematic bias to the estimated parameters. In this work, however, our simulations closely matched the in vivo observations in both correlations and variability, suggesting that the major confounds were controlled for adequately.

We observed an inverse relationship between a and κ in vivo, which might reflect a biological phenomenon or arise from model degeneracy. Importantly, κ may be influenced by non‐membrane‐related factors, such as capillarity or extracellular water, that are not modelled. Accurate DTI modeling is therefore essential for reliable parameter estimation, though this remains challenging in diseased muscle due to fat infiltration and fasciculations. Additionally, stronger signal attenuation of fat at long mixing times can further complicate RD estimation as a function of diffusion time [15, 60].

Although STE acquisitions at extended diffusion times are time‐consuming and can be clinically demanding, they remain promising for detecting subtle changes in muscle integrity. In this study, RD was estimated from a time‐efficient protocol (approximately 3 min per muscle region, two slices, four diffusion times). A standard DTI protocol with 30 volumes and 30 slices can also be acquired in a similar scan time. Improving model robustness and acquisition strategies, through optimized sampling or simultaneous multi‐slice acquisition, may enhance clinical utility. Further research should explore the use of RPBM in aging, muscle disease, and rehabilitation, while refining fitting algorithms to better disentangle the physiological origins of the fitted parameters.

## 6 Conclusion

In conclusion, this study provided a comprehensive assessment of the RPBM and its application in quantifying muscle microstructure in healthy individuals. Simulations and in vivo measurements demonstrated that cell diameter can be estimated robustly under appropriate model constraints, whereas membrane permeability remains sensitive to parameter selection and should be interpreted with caution. Cell diameter varies systematically with subject age, with significant differences between sexes and across muscle groups. Although isometric muscle force, measured as peak torque, also differed between sexes, no direct relationship between peak torque and cell diameter, permeability, FA, AD, or RD was found after accounting for sex, CSA, and age. Overall, these findings indicate that RPBM can complement traditional diffusion metrics, particularly in studies of muscle health, development, and pathology, provided that its modeling limitations are carefully considered.

## Author Contributions

MF: writing – original draft; writing – review and editing; methodology; conceptualization; funding acquisition; data curation; investigation; project administration; resources; software; formal analysis; visualization. RB: writing – review and editing; methodology; data curation; project administration; resources; investigation. DW: writing – review and editing; methodology; project administration; resources; investigation. LH: writing – original draft; writing – review and editing; data curation; project administration; resources; formal analysis; investigation. BB: writing – review and editing; methodology; project administration; resources.

## Funding

MF and LH are partly funded by the AES/TTW VIDI research programme (18929), which is financed by the Dutch Research Council (NWO).

## Conflicts of Interest

The authors declare no conflicts of interest.

## Supporting information


**Table S1:** Bonferroni‐corrected pairwise comparisons between muscle groups based on the linear mixed‐effects model. All six muscle groups were included as a fixed factor, with overall effects tested using Type III *F*‐tests and post hoc comparisons performed using estimated marginal means.

**Figure S1:** Distributions of RPBM‐derived parameters obtained for the forward model when D₀, τ, and ζ are all sampled from uniform distributions $D_{0}\in \left[1.0,2.0\right]$ μm^2^/ms, $\tau \in \left[100,900\right]$ ms, and $\zeta \in \left[0.5,2.0\right]$.

**Figure S2:** Parameter estimation accuracy for different fitting strategies using $t_{\text{full}}$ and constraining τ to a range of 350–750 ms. Scatter plots compare fitted versus true values for all model parameters across 5000 simulated signals using four fitting strategies (columns): all free, fixed $D_{0}$, and $D_{0}$ fixed incorrect. Rows show parameters $D_{0}$, τ, ζ, cell diameter a, and permeability κ. Red line indicates identity. The points are colored according to their value of $D_{0}$.

**Figure S3:** Parameter estimation accuracy for different fitting strategies using $t_{\text{clin}}$. Scatter plots compare fitted versus true values for all model parameters across 5000 simulated signals using four fitting strategies (columns): all free, fixed $D_{0}$, and $D_{0}$ fixed incorrect. Rows show parameters $D_{0}$, τ, ζ, cell diameter a, and permeability κ. Red line indicates identity. The points are colored according to their value of $D_{0}$.

**Figure S4:** Parameter estimation accuracy for different fitting strategies using $t_{\text{clin}}$ and constraining τ to a range of 350–750 ms. Scatter plots compare fitted versus true values for all model parameters across 5000 simulated signals using four fitting strategies (columns): all free, fixed $D_{0}$, and $D_{0}$ fixed incorrect. Rows show parameters $D_{0}$, τ, ζ, cell diameter a, and permeability κ. Red line indicates identity. The points are colored according to their value of $D_{0}$.

**Figure S5:** Relationship between in vivo DTI metrics (FA, AD, RD) and cell diameter for various diffusion times (black = men, *n* = 288, red = women, *n* = 282).

**Figure S6:** Relationship between in vivo DTI metrics (FA, AD, RD) and cell permeability for various diffusion times (black = men, *n* = 288, red = women, *n* = 282).

**Figure S7:** Relationship between cell diameter and permeability with biometric variables (CSA, age, weight, height, BMI). Each point represents a single muscle group per subject, and all six muscle groups are included. Separate linear fits are shown for men (black; *n* = 288), women (red; *n* = 282), and combined (blue) data.

**Figure S8:** Relationship between isometric extension torque and RBPM‐derived parameters in the Quadriceps, Hamstrings, and Adductors. Linear trends are shown for men (black; *n* = 48), women (red; *n* = 47), and combined (blue) subjects.

## Data Availability

The data that support the findings of this study are available from the corresponding author upon reasonable.
