# Supplementary material for: Modeling, Evaluation, and In Vivo Estimation of Muscle Cell Diameter With the Random Permeable Barrier Model: Correlation With Subject Characteristics and Isometric Torque
Source: NMR Biomed. 2026 Jan 20;39(3):e70233. doi: 10.1002/nbm.70233 (PMC12818024; doi:10.1002/nbm.70233)
Supplement: Supplementary file 1 — Table S1: Bonferroni‐corrected pairwise comparisons between muscle groups based on the linear mixed‐effects model. All six muscle groups were included as a fixed factor, with overall effects tested using Type III F‐tests and post hoc comparisons performed using estimated marginal means. [file NBM-39-e70233-s009.docx]

| Supplemental Table T 1: Bonferroni-corrected pairwise comparisons between muscle groups based on the linear mixed-effects model. All six muscle groups were included as a fixed factor, with overall effects tested using Type III F-tests and post hoc comparisons performed using estimated marginal means. | | | | | | | |
| --- | --- | --- | --- | --- | --- | --- | --- |
|  | **CSA [cm^2^]** | **α [µm]** | **κ [µm/s]** | **FA [-]** | **AD [µm^2^/ms]** | **RD [µm^2^/ms]** |  |
| **Adductors -  Anterior** | ***<0.001*** | ***<0.001*** | ***<0.001*** | ***<0.001*** | ***<0.001*** | ***<0.001*** |  |
| **Adductors -  Deep post.** | ***<0.001*** | ***<0.001*** | ***<0.001*** | ***<0.001*** | ***<0.001*** | ***<0.001*** |  |
| **Adductors -  Hamstrings** | ***<0.001*** | ***<0.001*** | ***<0.001*** | ***<0.001*** | ***<0.001*** | ***<0.001*** |  |
| **Adductors -  Quadriceps** | ***<0.001*** | ***0.014*** | 1.000 | 0.482 | 1.000 | 1.000 |  |
| **Adductors -  Superf. post.** | ***<0.001*** | 0.414 | ***<0.001*** | ***0.039*** | 1.000 | 0.059 |  |
| **Anterior -  Deep post.** | 1.000 | 0.055 | ***<0.001*** | ***0.001*** | ***0.026*** | ***<0.001*** |  |
| **Anterior -  Hamstrings** | ***<0.001*** | ***<0.001*** | ***0.037*** | ***<0.001*** | 0.644 | ***0.006*** |  |
| **Anterior -  Quadriceps** | ***<0.001*** | ***<0.001*** | ***<0.001*** | ***<0.001*** | ***0.020*** | ***0.047*** |  |
| **Anterior -  Superf. post.** | ***<0.001*** | ***<0.001*** | ***0.017*** | ***<0.001*** | ***<0.001*** | ***<0.001*** |  |
| **Deep post. -  Hamstrings** | ***<0.001*** | 0.518 | ***<0.001*** | 0.676 | ***<0.001*** | 1.000 |  |
| **Deep post. -  Quadriceps** | ***<0.001*** | ***0.002*** | ***<0.001*** | ***0.007*** | ***<0.001*** | 1.000 |  |
| **Deep post. -  Superf. post.** | ***<0.001*** | ***<0.001*** | ***<0.001*** | ***<0.001*** | ***<0.001*** | ***<0.001*** |  |
| **Hamstrings -  Quadriceps** | ***<0.001*** | ***0.003*** | ***<0.001*** | ***0.013*** | ***0.038*** | 0.791 |  |
| **Hamstrings -  Superf. post.** | ***<0.001*** | ***<0.001*** | 0.511 | ***<0.001*** | ***<0.001*** | ***<0.001*** |  |
| **Quadriceps -  Superf. post.** | ***<0.001*** | 0.066 | ***<0.001*** | ***<0.001*** | 1.000 | ***<0.001*** |  |
