# Supplementary figures and images for: Modeling, Evaluation, and In Vivo Estimation of Muscle Cell Diameter With the Random Permeable Barrier Model: Correlation With Subject Characteristics and Isometric Torque
Source: NMR Biomed. 2026 Jan 20;39(3):e70233. doi: 10.1002/nbm.70233 (PMC12818024; doi:10.1002/nbm.70233)

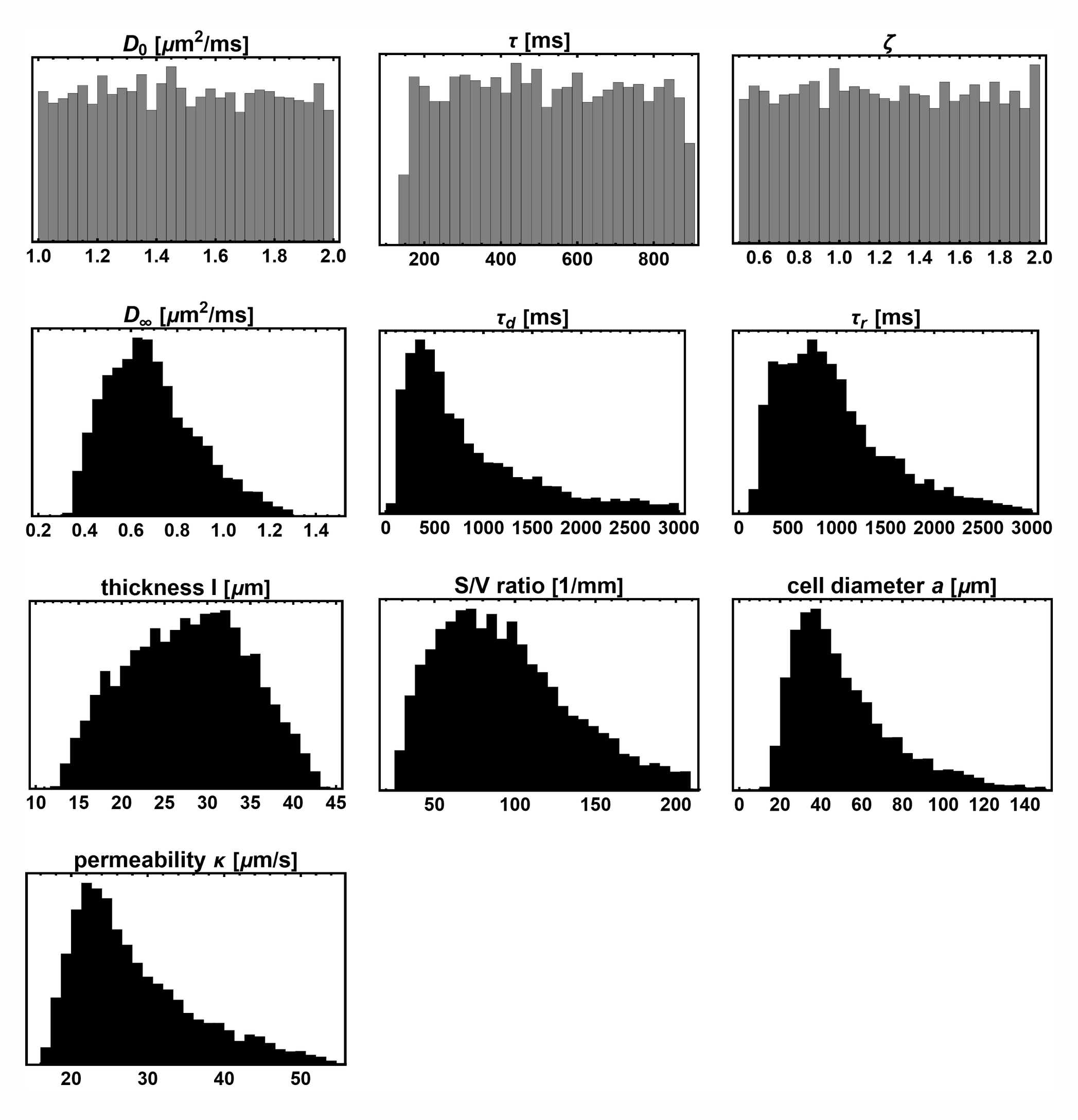

Supplement: Supplementary file 2 — Figure S1: Distributions of RPBM‐derived parameters obtained for the forward model when D₀, τ, and ζ are all sampled from uniform distributions D0∈1.0,2.0 μm2/ms, τ∈100,900 ms, and ζ∈0.5,2.0. [file NBM-39-e70233-s006.png]

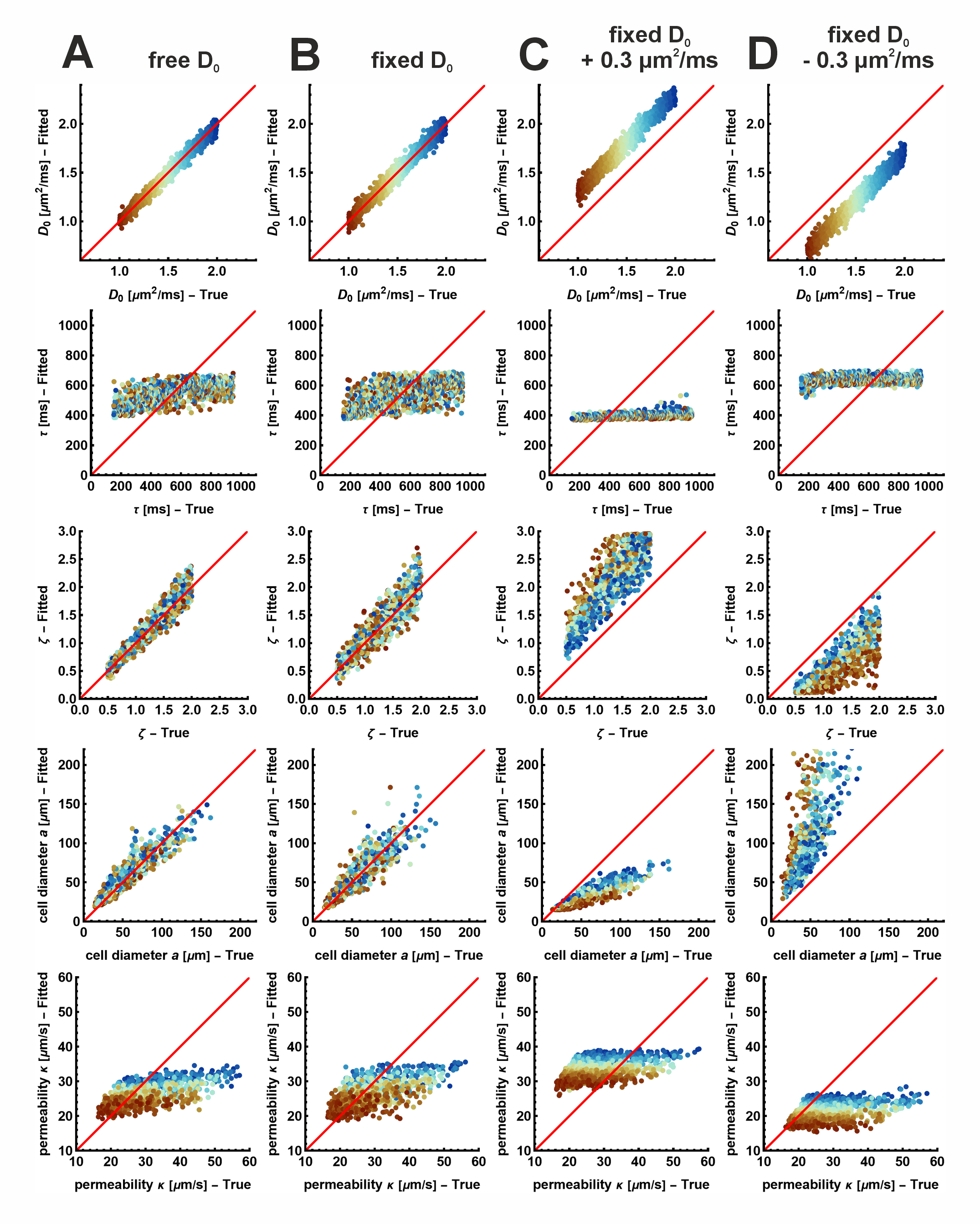

Supplement: Supplementary file 3 — Figure S2: Parameter estimation accuracy for different fitting strategies using tfull and constraining τ to a range of 350–750 ms. Scatter plots compare fitted versus true values for all model parameters across 5000 simulated signals using four fitting strategies (columns): all free, fixed D0, and D0 fixed incorrect. Rows show parameters D0, τ, ζ, cell diameter a, and permeability κ. Red line indicates identity. The points are colored according to their value of D0. [file NBM-39-e70233-s003.png]

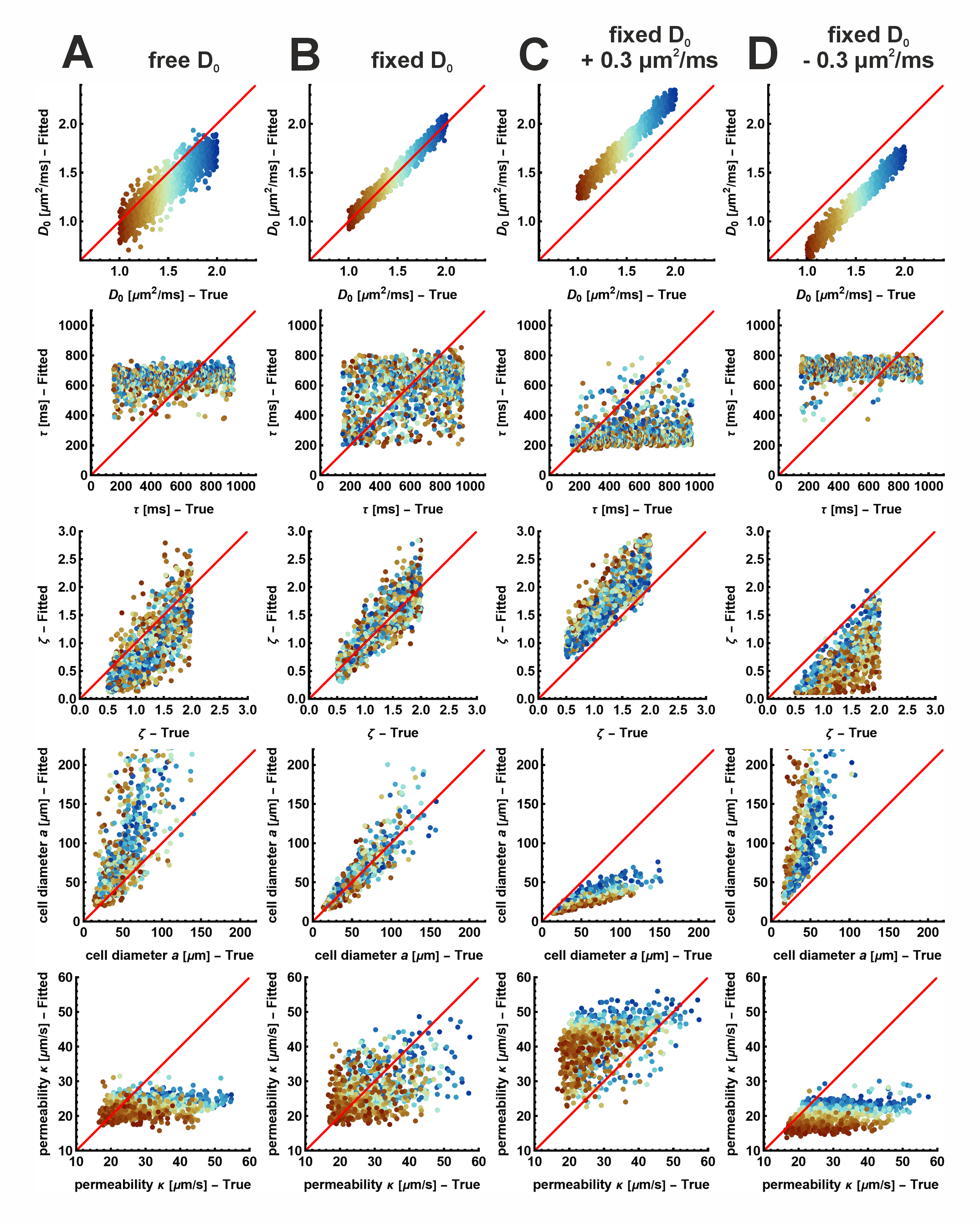

Supplement: Supplementary file 4 — Figure S3: Parameter estimation accuracy for different fitting strategies using tclin. Scatter plots compare fitted versus true values for all model parameters across 5000 simulated signals using four fitting strategies (columns): all free, fixed D0, and D0 fixed incorrect. Rows show parameters D0, τ, ζ, cell diameter a, and permeability κ. Red line indicates identity. The points are colored according to their value of D0. [file NBM-39-e70233-s007.png]

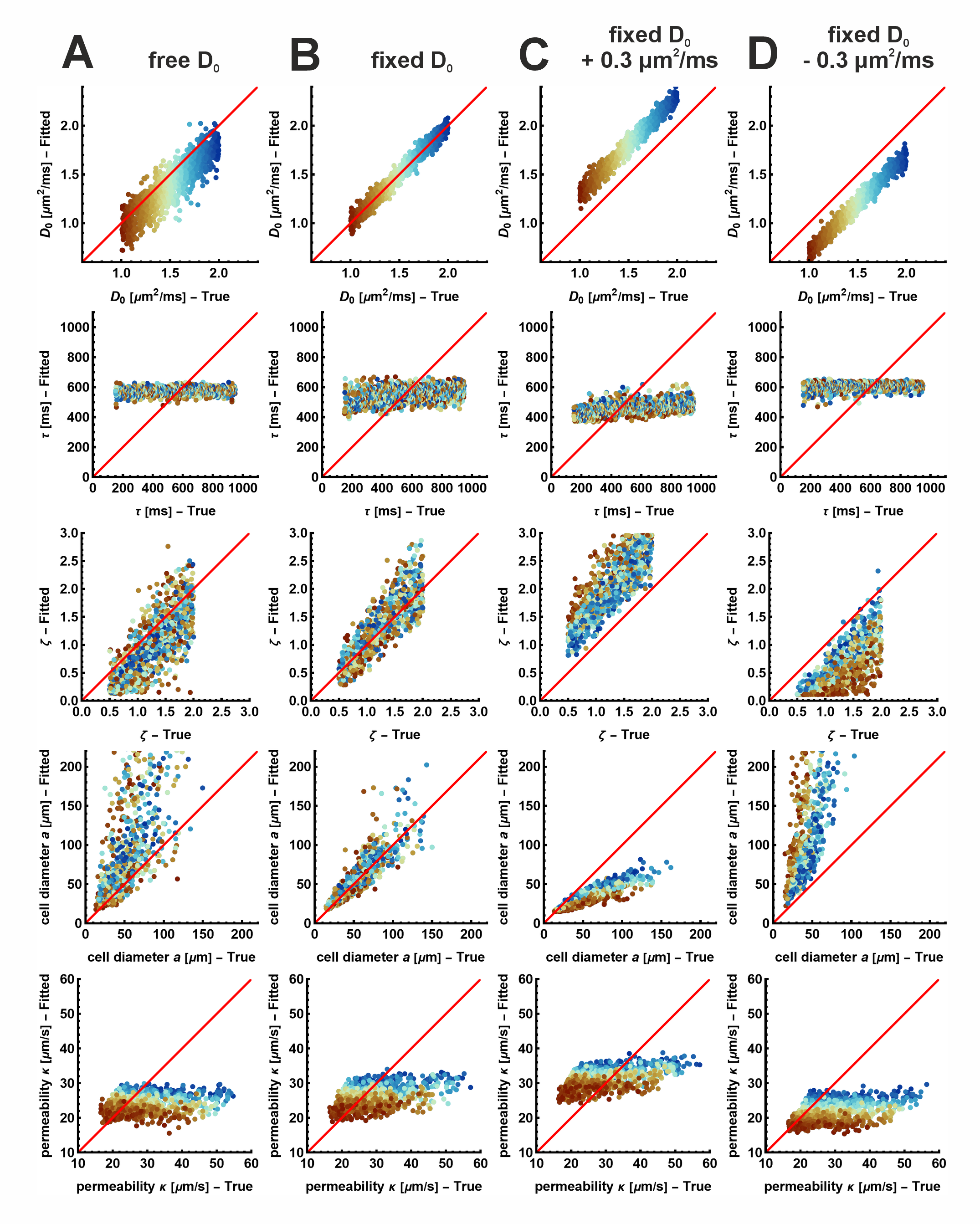

Supplement: Supplementary file 5 — Figure S4: Parameter estimation accuracy for different fitting strategies using tclin and constraining τ to a range of 350–750 ms. Scatter plots compare fitted versus true values for all model parameters across 5000 simulated signals using four fitting strategies (columns): all free, fixed D0, and D0 fixed incorrect. Rows show parameters D0, τ, ζ, cell diameter a, and permeability κ. Red line indicates identity. The points are colored according to their value of D0. [file NBM-39-e70233-s008.png]

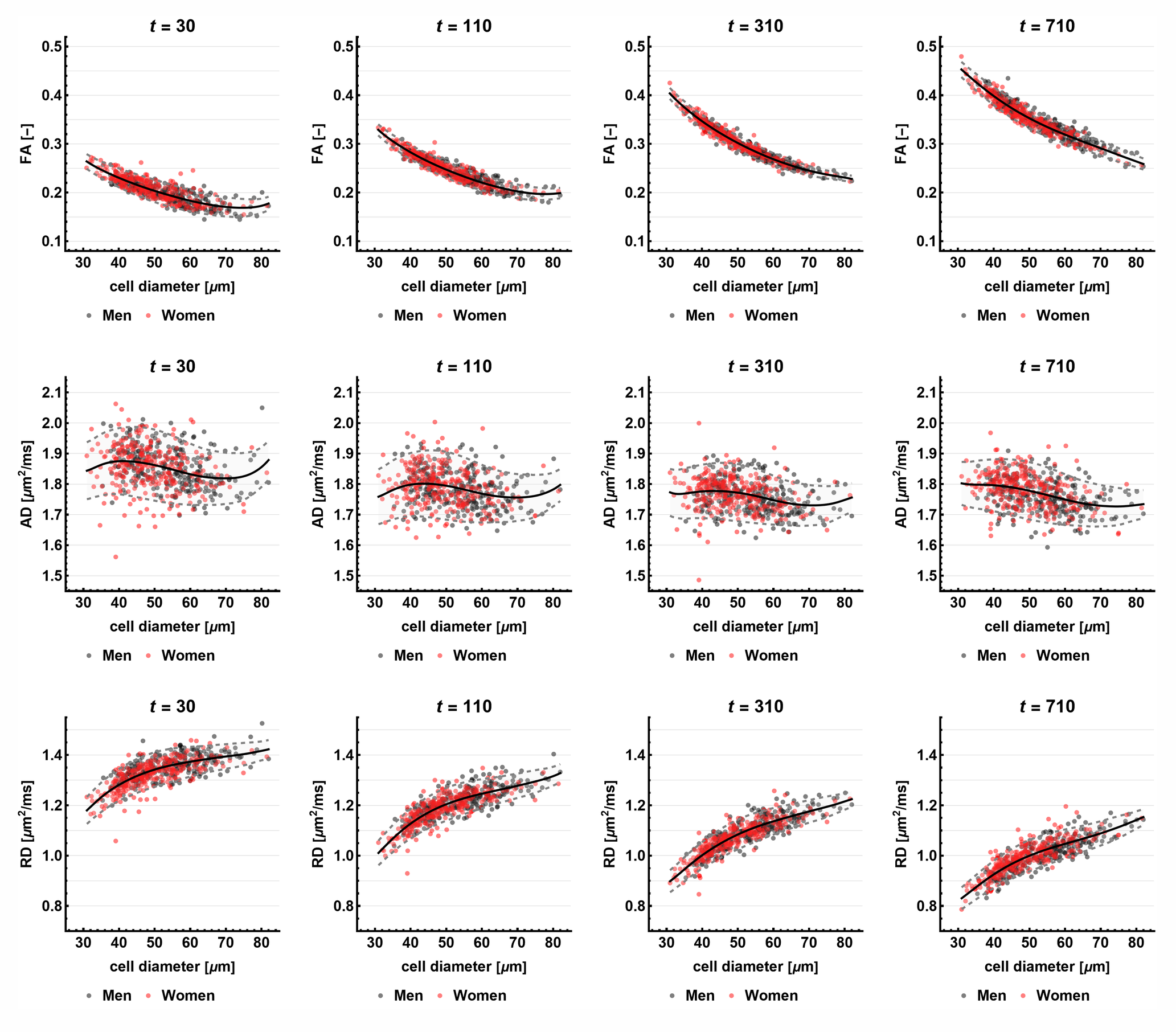

Supplement: Supplementary file 6 — Figure S5: Relationship between in vivo DTI metrics (FA, AD, RD) and cell diameter for various diffusion times (black = men, n = 288, red = women, n = 282). [file NBM-39-e70233-s001.png]

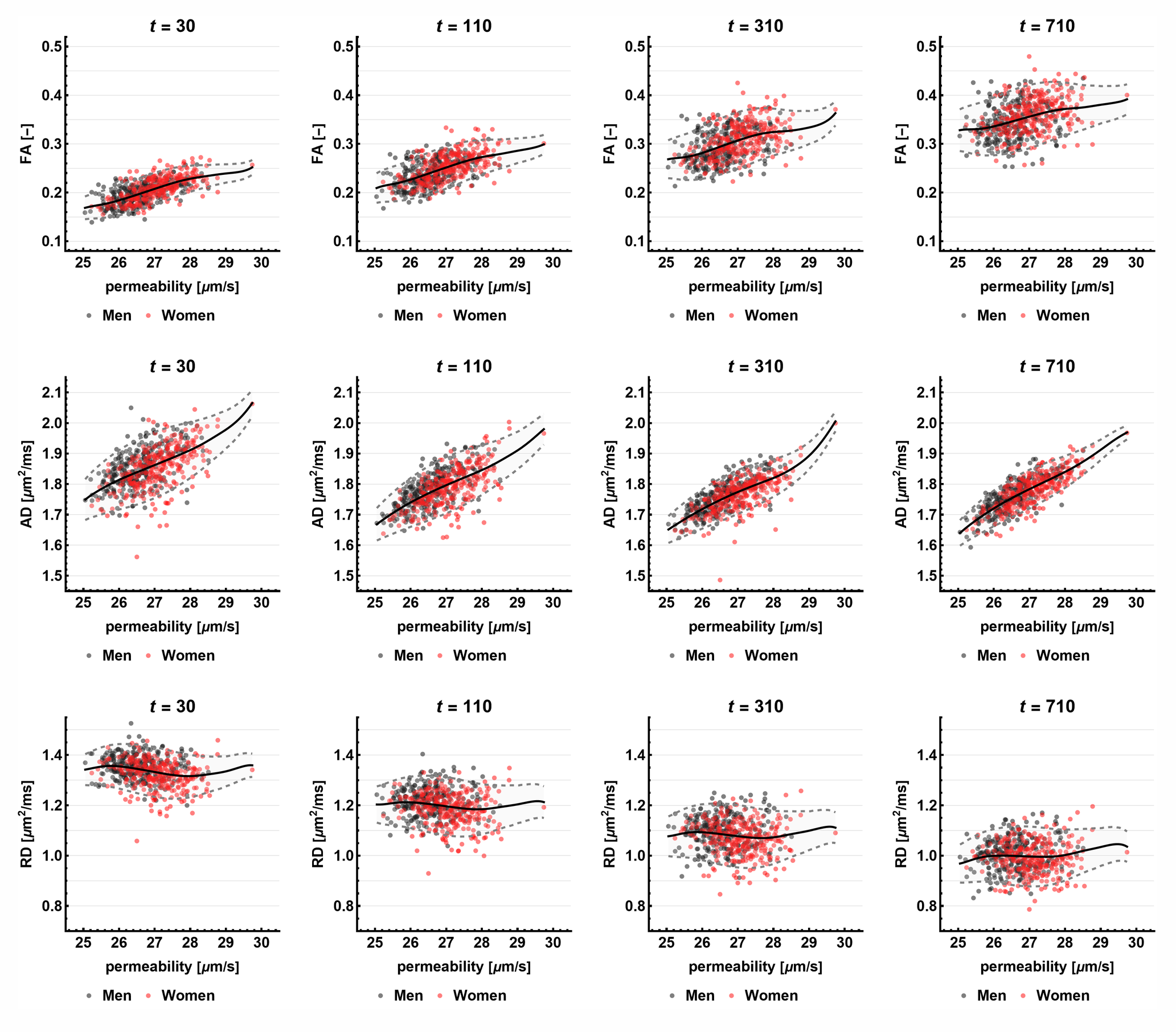

Supplement: Supplementary file 7 — Figure S6: Relationship between in vivo DTI metrics (FA, AD, RD) and cell permeability for various diffusion times (black = men, n = 288, red = women, n = 282). [file NBM-39-e70233-s005.png]

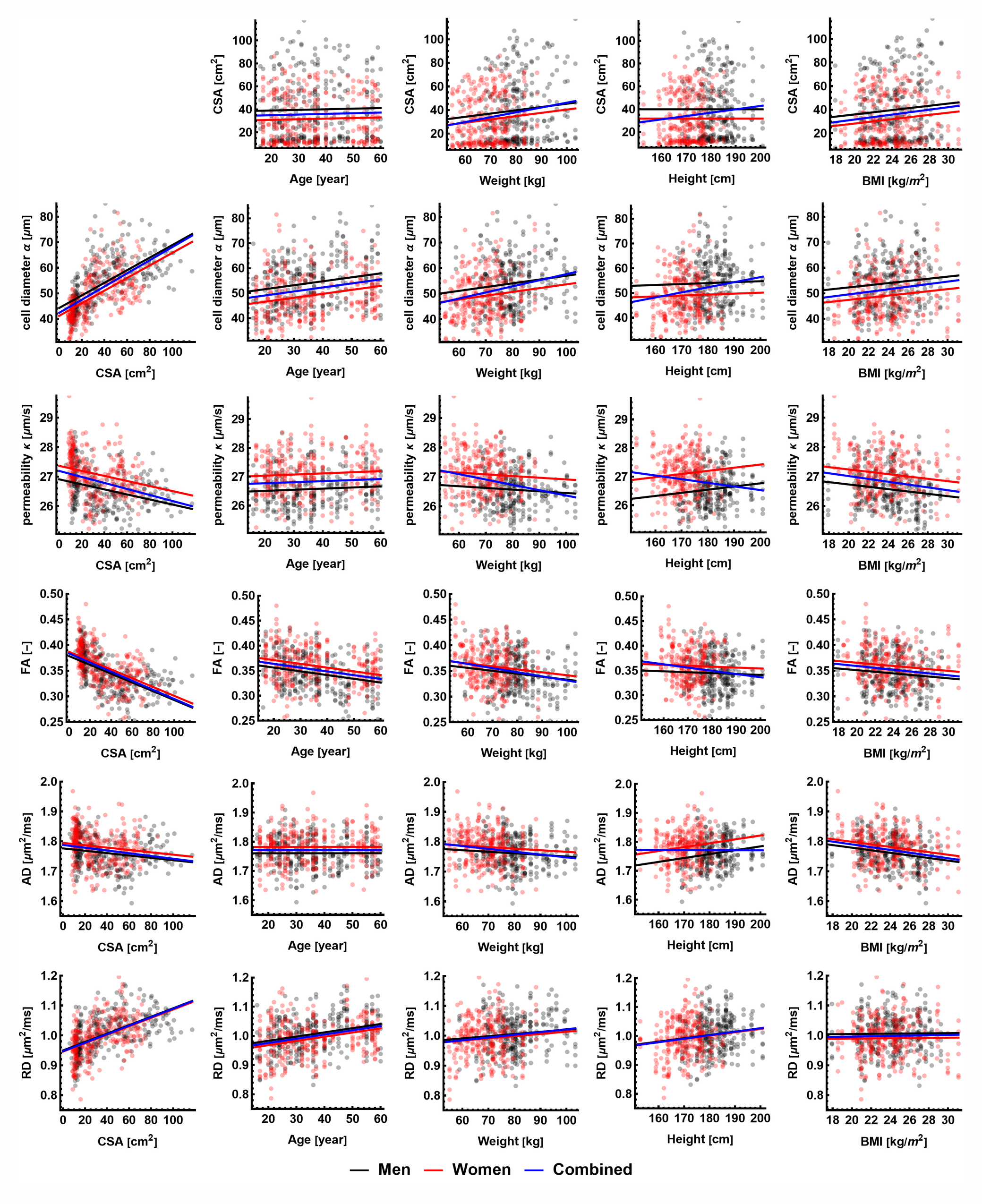

Supplement: Supplementary file 8 — Figure S7: Relationship between cell diameter and permeability with biometric variables (CSA, age, weight, height, BMI). Each point represents a single muscle group per subject, and all six muscle groups are included. Separate linear fits are shown for men (black; n = 288), women (red; n = 282), and combined (blue) data. [file NBM-39-e70233-s004.png]

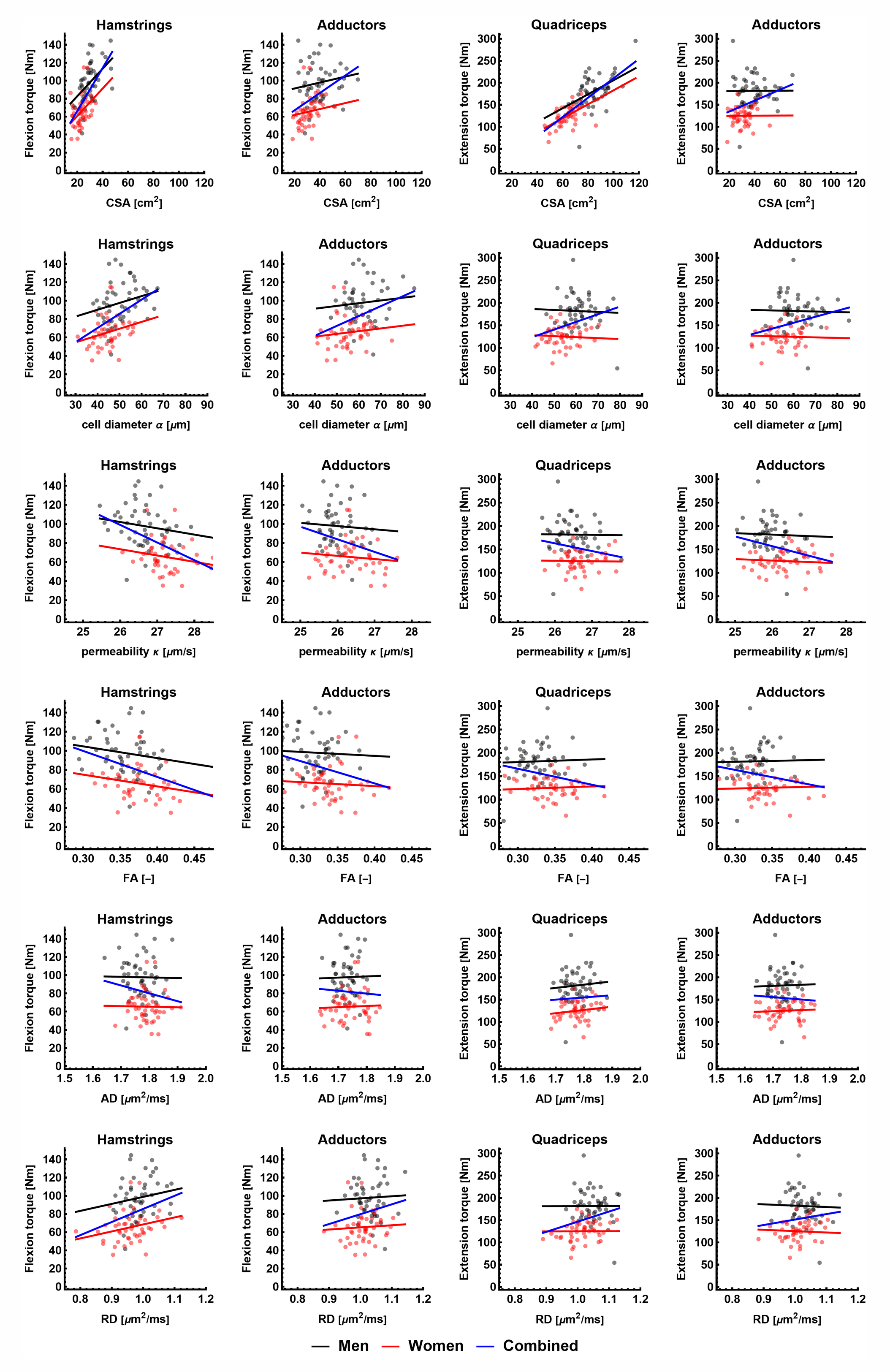

Supplement: Supplementary file 9 — Figure S8: Relationship between isometric extension torque and RBPM‐derived parameters in the Quadriceps, Hamstrings, and Adductors. Linear trends are shown for men (black; n = 48), women (red; n = 47), and combined (blue) subjects. [file NBM-39-e70233-s002.png]
